# The experience of young women with gestational syphilis from the conceptual perspective of hope

**DOI:** 10.1590/1980-220X-REEUSP-2025-0118en

**Published:** 2026-07-24

**Authors:** Monika Wernet, Zaida Charepe, Constança Festas, Aline Oliveira Silveira, Patrícia Akari Nakao

**Affiliations:** 1 Universidade Federal de São Carlos, Departamento de Enfermagem, São Carlos, SP, Brazil.; 2 Universidade Católica Portuguesa, Faculdade de Ciências da Saúde e Enfermagem, Lisboa, Portugal.; 3 Universidade de Brasília, Departamento de Enfermagem, Brasília, DF, Brazil.; 4 Universidade Federal de São Carlos, Programa de Pós-graduação em Enfermagem, São Carlos, SP, Brazil.

**Keywords:** Syphilis, Delivery of Health Care, Hope, Prenatal Care, Professional-Patient Relations

## Abstract

**Objective:**

To understand the hope of young women diagnosed with gestational syphilis in the context of prenatal care.

**Method:**

Qualitative research, based on thematic analysis, from the conceptual perspective of hope. It was developed in the state of São Paulo with young women diagnosed with gestational syphilis during prenatal care through semi-structured interviews and audio recordings conducted between July 2023 and April 2024.

**Results:**

Sixteen young women participated in the study, all self-identifying as black or mixed-race, four of whom were first-time mothers, and most had up to nine years of schooling. The experience of hope is portrayed in the following thematic units: “Threats to hope”, with the subtopics “Stigma and suffering” and “Restricted informational and social support”; and “Promoters of hope”, with the subtopics “Child protection” and “Self-preservation”.

**Conclusion:**

In prenatal care, women faced a dialectic of threatening and hope-promising contexts. In this sense, the following stood out, respectively: the incomplete professional support for emotional and informational needs, and the clarity of the desired objective (non-contamination of their child) associated with internalized reflection on the exercise of their maternal role.

## INTRODUCTION

There is a growing body of evidence on hope in healthcare and nursing, all highlighting the contributions of this construct to coping with adverse, threatening, and/or complex situations. Hope leads to the identification of goals and to the meaning of experiences, being highly personal^(1,2)^.

The incorporation of hope into clinical practice favors holistic and comprehensive care^(1,2,3)^, centered on the person, with recognition of the uniqueness of suffering, difficulties, needs, and coping processes^(2,3)^. It promotes a transformative force, with positive expectations, motivation, and inner willingness to establish and achieve goals^(2,3,4)^. The process of hope is related to determination (willpower) and behaviors (waypower) to achieve something intended^(5)^, a hoped-for object.

In the presence of hope, behaviors of appreciation and dynamic, continuous assessment of the situation and available resources in relation to the desired object occur, with the establishment of strategies to achieve it^(2,6,7)^. This promotes resilience and enables overcoming adversities, with effects on comfort^(6)^, quality of life, well-being^(7)^, health^(6)^, and life. High levels of hope lead individuals to adopt, in the present, behaviors directed toward future goals^(6)^.

When considering the encounter for the provision of healthcare and nursing care, professional behavior is a determinant of hope^(2,3,4)^, an aspect that implies assuming commitments toward it, considering it in interventions^(3)^. Interpersonal and intrapersonal relationships are among the determinants of hope, shaping its manifestation^(4,6)^.

Evidence related to hope is concentrated in the field of palliative care and chronic illness, addressing different life stages and health-disease transitions^(1)^. There are gaps in discussions of hope in the context of a syphilis diagnosis, an infectious disease that constitutes a global public health problem and challenge. Gestational syphilis (GS), specifically, is associated with a higher risk of spontaneous abortion, preterm birth, and occurrence of congenital syphilis, all threats to life and health, with implications for maternal and child morbidity and mortality^(8,9)^.

According to the 2024 Epidemiological Bulletin of the Brazilian Ministry of Health, in 2023, GS cases were approximately 34 per 1,000 live births, totaling 86,111 notifications, an 11% increase compared to the rate analyzed in the previous year^(9)^. In this scenario, adolescents and young people stood out as population groups with higher occurrence of the disease^(9)^, making it urgent to explore discussions specific to these groups.

The control of GS has been a challenge. Scientific evidence and clinical approaches are strongly guided by risk epidemiology, with maximum efforts toward early diagnosis, timely treatment of women, and reaching their sexual partners for diagnosis and treatment^(8,9)^, which are important intervention measures. Despite advances, the disease shows reemergence in Brazil and worldwide^(8,9,10)^, calling for innovations in healthcare. In this direction, equitable access to healthcare services and quality prenatal care is pointed out as having an impact on addressing this situation^(11)^ and as a guarantee of rights.

In Brazil, the history of syphilis and public health campaigns directed at it involves discursive and symbolic constructions embedded in moral judgments, especially regarding sexual practices^(12)^. Furthermore, there is a strong emphasis on individual behavior, structured within a biomedical, hygienist, and public health logic^(8,9,10)^.

There is an urgent need, in responding to the reemergence of the disease, to go beyond the aforementioned approach, considering social, cultural, and subjective issues^(12,13)^, as well as generational characteristics. Social labeling may extend to individuals’ self-perception and affect coping processes^(13)^, including in the context of hope.

The present study questions the experience of hope among young women diagnosed with GS in the context of prenatal care. It is based on the assumption that this focus may provide indications for renewing healthcare practices and those of professionals, contributing to a shift in the approach to care encounters related to the disease, considering subjectivities and social determinants present in the context. The objective was to understand the hope of young women diagnosed with GS in the context of prenatal care.

## METHOD

### Study Design

This is a qualitative, descriptive, and exploratory study, guided by the Consolidated criteria for Reporting Qualitative research^(14)^, grounded in the methodological framework of thematic analysis^(15)^ and the Hope Model of Dufault and Martocchio^(3)^. A secondary analysis of research data was conducted with young women diagnosed with GS during prenatal care regarding their perception of the healthcare received.

Hope encompasses two spheres, generalized and particularized, both with the same six dimensions: (1) affective, encompassing the emotions and feelings involved with the intended hoped-for object; (2) cognitive, encompassing thoughts and desires related to the hoped-for object and involving the process of identifying it and evaluating reality for its achievement; (3) behavioral, referring to action and orientation toward producing what is desired; (4) affiliative, linked to relationships with oneself, with others, and with the social environment; (5) temporal, involving the recognition that hope is directed toward the future but depends on present and past experiences; and (6) contextual, reflecting the consideration and relevance of the social context in which one is embedded^(3)^. Generalized hope differs from particularized hope in that the intended future is uncertain. In particularized hope, there is a defined hoped-for object toward which investments are made. The latter acts in constructive coping^(3)^.

Furthermore, hope is an existential process, shaping feelings, behaviors, and relationships with oneself and others. It emerges as a process in and from the interaction of four attributes: transcendence (the soul of hope); experiential (the pain of hope); relational (the heart of hope); and rational (the mind of hope)^(16)^.

### Setting, Population, and Selection Criteria

The study was conducted in a Brazilian city located in the countryside of the state of São Paulo. The selection criteria for participation in the study were: being a woman, being emancipated, aged between 15 and 24 years, and having a diagnosis of syphilis during pregnancy. Those who were not in psychological condition to participate in the narrative interview were excluded.

Participants were recruited from a maternity hospital in the central-eastern region of the state of São Paulo, a regional reference for pregnancy, childbirth, and the postpartum period. Belonging to Regional Health Department III, the maternity hospital recorded 1,991 births in 2023 and 1,924 in 2024, of which approximately 7% were babies with GS. Of these, slightly more than half were aged between 15 and 24 years, according to service management information. According to *Instituto Brasileiro de Geografia e Estatística*, in 2022, the city had a population of 254,857 inhabitants, of which 6.73% were women aged between 15 and 24 years^(17)^.

### Data Collection

Data collection took place between July 2023 and April 2024 through audio-recorded interviews conducted in participants’ homes. The invitation to participate in the study was made by the maternity hospital’s nurse manager. Upon acceptance, she mediated contact with the research team, obtaining the participant’s phone number and requesting authorization to share it. Subsequently, the researchers contacted the participants to schedule the interview. On the day of the interview, the researchers revisited the study objectives, confirmed the participants’ consent, and then proceeded with signing the Informed Consent Form (ICF). It is noteworthy that excerpts included in this manuscript were identified with the letter “W”, referring to the word “woman”, followed by an Arabic numeral indicating the order of inclusion in the study.

The first part of the interview focused on characterizing participants in terms of age, marital status, race, gestational trimester at the time of syphilis diagnosis, and gestational and parity data. The second part included a prompt aimed at recalling “when the diagnosis of syphilis was received and how it occurred and how care was perceived”. Additional questions to deepen the perception of prenatal care were presented according to the participant’s narrative.

The interviews lasted between 18 and 35 minutes. They were conducted by the last author, a researcher in training, under the supervision and support of the first author, who has experience in conducting qualitative studies using interviews. Field notes were taken regarding contextual details to contribute to data triangulation and to the interpretation of transcribed interviews. Likewise, analyses and data were shared in meetings of the Hope2Care research group to incorporate different perspectives. This group, led by the second author of this manuscript, includes research, intervention, and training projects related to hope in health-disease processes. Partial results were presented and discussed in two group meetings, maintaining participant anonymity.

The criterion of thematic saturation determined the end of the interviews, i.e., the dataset obtained and its analysis reached recurrence of codes or topics. No new information or relationships emerged in the understanding of the study object^(18)^.

### Data Analysis

In order to understand the experience of hope among women, the reanalysis of interviews from the primary study was guided by considerations regarding the dimensions and spheres of hope^(3)^ and its process^(16)^.

The transcribed interview texts were analyzed by the first author under the supervision of the second author of this manuscript. The analytical process was conducted using thematic analysis^(15)^, structured in six stages. The first stage consisted of repeated readings to identify meanings and patterns associated with the object of study—hope in the context of prenatal care among young women with GS—with the selection of representative excerpts (second stage, selection of key elements). These excerpts were highlighted considering the dimensions of hope^(3)^, its process, and attributes^(16)^. Efforts were directed toward understanding hope, how it manifested and connected to the woman’s existence, as well as the emotions, feelings, and thoughts shaped by the perception of the social context. Furthermore, how this whole was articulated with the achievement of the hoped-for object guided the selection and analysis of excerpts. These excerpts were examined to understand the meanings of words and linguistic constructions. In other words, keywords were identified and subsequently coded (third stage, code selection). These codes were grouped into topics (fourth stage), followed by new inductive analytical processes aimed at identifying patterns and relationships (fifth stage, conceptualization), culminating in the development of the conceptual model, corresponding to the final stage of thematic analysis. The resulting conceptual model was structured into two main topics: one related to the promotive dimension and another related to the limiting dimension of the experience of hope.

### Ethical Aspects

The study followed the ethical principles established by the Brazilian National Health Council for research involving human beings. It was approved by a Research Ethics Committee involving human subjects, under Opinion 6,200,614 and Certificate of Presentation for Ethical Consideration 69634923. 1.0000.5504. Participants signed the ICF.

## RESULTS

Twenty-three women were invited to participate in the study, of whom three declined due to reporting that discussing the situation caused them significant discomfort. Of the 20 women who agreed to participate, four did not respond to two contact attempts to schedule the interview and were considered dropouts. Thus, 16 women were interviewed, with a mean age of 18 years. Two were emancipated, and three had a history of reinfection with *Treponema pallidum*. All women self-identified as black or mixed-race. In addition, four were primiparous, six were multiparous (second pregnancy), 11 had up to nine years of schooling, and one was a single mother.

The experience of hope among young women diagnosed with GS in the context of healthcare occurs through the dialectical management of threatening aspects and promoters of hope, as shown in Charts 1 and 2.

**Chart 1:** Codes and subtopics of the topic “Threats to hope” – São Carlos, SP, Brazil, 2025.

Initial codes	Intermediate codes	Subtopic
Perceptions of stigma	Stigma and prejudice	Stigma and suffering
Experiences of violence		
Not disclosing oneself		
Discomforts	Emotional overload	
Guilt		
Fears and worries		
Doubts and lack of knowledge	Lack of knowledge	Restricted informational and social support
Internet use		
Sharing one’s condition and social support	Limited social support network	
Loneliness		
Topic: Threats to hope		

**Chart 2:** Codes and subtopics of the topic “Promoters of hope” – São Carlos, SP, Brazil, 2025.

Initial codes	Intermediate codes	Subtopic
Belief in divine protection	Beliefs	Child protection
Belief in positive outcomes		
Exams	Adherence to professional recommendations	
Treatment		
Going to appointments		
Interaction with the child	Self-monitoring	Self-preservation
Thoughts about oneself in the role of mother		
Breakdown of relationships	Respecting their limits	
Reflections on humiliations		
Topic: Threats to hope		

**Figure 1:**
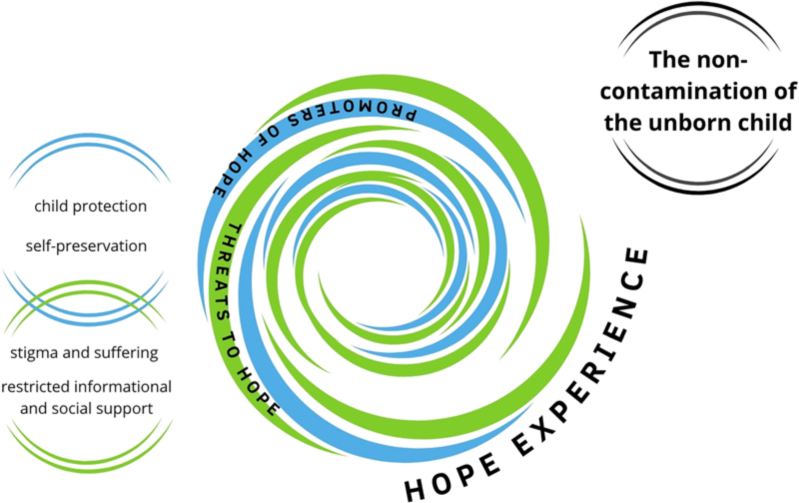
Graphic synthesis of the conceptual model “Experience of hope of young women with gestational syphilis in healthcare”. São Carlos, SP, Brazil, 2025.

In the context of relationships and communication with healthcare professionals during prenatal care, participants experienced the main threats to their hope. However, they needed to expose themselves to these threats in order to achieve the object of their hope: preventing contamination of the child they were carrying. Healthcare professionals were the ones who ensured access to treatment for GS and diagnostic tests. By reaffirming their engagement with syphilis treatment, the women felt they were protecting their child, which was central to the projection and strengthening of hope. The topics “Threats to hope” and “Promoters of hope”, with their respective subtopics, detail the experience of hope among young women diagnosed with GS in the context of healthcare and are summarized in Figure 1.

*Topic: Threats to hope*

*Subtopic: Stigma and suffering*

The hope of the woman, as the mother of a child exposed to *Treponema pallidum* during pregnancy, is constructed within a relational context immersed in communications that suggest prejudice and generate feelings such as shame, discomfort, and distress.

From the woman’s perspective, when interacting with the symbol “GS”, the professional activates the meaning of harm to the child and begins to emphasize, throughout the interaction, that the bacterium is transmissible via the uteroplacental route and may have severe consequences for the child, making it her duty to follow the prescribed treatment. In this relational context, professionals’ communications (verbal, nonverbal, and paralinguistic) convey symbols of judgment. The recurrence and intensity of these communications reverberate as moral violence, leading the woman to avoid exposing herself in interactions with professionals.

*There is a lot of judgment. They* (maternity professionals) *whisper among themselves and you notice everything.* [...] *I remember when I took the test and it came back positive* (for syphilis). *The nurse widened her eyes and immediately started asking if I knew what it was, saying it was a sexually transmitted disease, that I had to take care of myself, that I was young, that it would harm him* (the unborn child). *She talked a lot. I felt ashamed, I wanted to run away; I just kept looking down.* (W16)

Interactions in the healthcare context generated discomfort, to the point that some considered discontinuing their attendance at healthcare services to avoid exposure.

*During prenatal care, I had to go through a lot of prejudice about syphilis as a disease of people who sleep around.* [...] *I got it from her father. It hurts to hear these things,* [...] *it brings shame, a lot of shame.* [...] *sometimes it is so much that I think about not coming; I come not wanting to come.* (W5)

*They look at you and prejudice already comes with the word “syphilis”* (she thinks). [...] *the atmosphere of the conversation is very unpleasant and causes discomfort.* (silence) *then you go in and out always as quietly as possible. You answer only what is necessary and leave quickly. I always feel anxious about coming.* [...] *I don’t like coming because I already know how it will be*. (W10)

Thus, they felt guilty for thinking that their child had been or could be at risk due to GS and suffered because of it.

*The first pregnancy is a unique moment. And then a disease like this! My goodness, it hit me hard. But I followed the treatment, all the tests. I did everything properly; we cannot neglect it, right? It is our health, not only ours but also the baby’s.* [...] *I cried, and how I cried... alone. I still cry when I think that the baby could become blind or have developmental issues because of me.* (W13)

The process of analyzing the evidence in light of their lived experience resulted in emotional overload and moral exhaustion, another element that threatened hope.

### Subtopic – Restricted Informational and Social Support

Being subjected to relationships within healthcare services permeated by prejudice reduces women’s chances of expanding and qualifying the information they have about syphilis. They did not feel encouraged to ask questions, fearing exposure to judgmental comments. All participants in this study reported gaps in their knowledge about syphilis and stated that they resorted to the internet to address them. They avoided raising questions during interactions with professionals due to fear of discomfort. Thus, knowledge to understand their situation and generate hope was built based on limited information about syphilis.

*I didn’t have knowledge* (about the disease). *Even today, I don’t really understand it properly. When they told me* (about the disease), *I was scared. I searched, I went to look up what it was, and then I read that if you undergo treatment, everything will be fine. So, I felt reassured because I did the treatment, but even today I don’t understand whether the treatment removes the disease from the body or if it never really goes away. It seems like it stays, right? It never goes away, right? And in the baby too?* (W10)

In addition, aware of the socially widespread meaning of syphilis, they disclosed their condition to very few people within their social network. They almost treated it as a secret, usually shared only with a partner and/or a close family member, especially the mother. One participant even chose to “forget” the situation:

*I was afraid I had passed it to her* (the child), *but I took six doses of the medication. I had a blood test and it was reactive, then I repeated it and it was no longer reactive, it showed only a small amount, so we decided* (she and her partner) *to forget about it. We are not going to look into it anymore and we didn’t tell anyone* [...] *we decided not to tell anyone and to forget, erase it* [...] (W1)

Given this context, the experience became almost solitary, with the woman herself, through internal reflections, as the main support in dealing with doubts, fears, and concerns. It is noteworthy that, for one participant, not seeking information beyond what professionals had told her was a strategy to cope.

*I was afraid, worried... it affects your psychological state. I was angry, I was afraid it could affect him somehow. I didn’t look for anything else, so I wouldn’t get more distressed.* (W4)

### Topic: Promoters of Hope

The experience of hope is sustained by the protection of oneself and of the child. To this end, women sought to preserve themselves and ensure the best possible performance in their maternal role.

### Subtopic: Child Protection

Interactions with healthcare professionals, from the moment of diagnosis of GS, promoted the understanding that syphilis is monitored through tests and that treatment is the action that ensures “cure” or the non-contamination of the unborn child. Thus, all participants adhered to treatment and underwent tests according to professional recommendations, which were experienced as symbols of maternal care. In this context, amid uncertainties, they appealed to divine protection.

[...] *what became stronger was not passing it to her... that’s what I valued, I thought about her, about not passing it on.* [...] *then I started thinking about her and everything I had to do, and they said it was the treatment and doing the tests to see if it worked. And I did it. So, what helped was thinking about her and believing it would work, because I was doing everything they told me.* (W1)

*I did everything properly, just as they told me. I hope it works out and that he doesn’t suffer and doesn’t have anything.* (W7)

*There were only a few days left between finishing the medication and her birth. I’m a bit nervous about whether it will work. I got a little discouraged, I made so much effort, endured all those injections, very painful. It cannot happen that she has the disease. God willing, it won’t. God will protect her; I believe in Him* [...] (W11)

Among women who had experienced syphilis in a previous pregnancy, interaction with that child contributed to coping. Having prior experience supported decision-making in the current situation. Moreover, belief in divine protection strengthened hope.

*Believing in God, I prayed a lot. Every night I prayed for his health. I’m very religious. I go to church every week and always asked for his health. And it made me see that it helped* (thinks)... *I think looking at my other child and seeing that it worked out with him.* (W2)

### Subtopic: Self-Preservation

The emotional burden experienced in interactions with professionals triggered an intense process of internal reflection, through which women reaffirmed their actions to minimize the transmission of syphilis to the child and recognized their efforts to ensure what was within their reach. These internal dialogues guided decisions related to social exposure, especially due to the stigma associated with the disease. Thus, they sought to protect themselves in order to endure humiliation, guilt, and concerns, sustaining their hope. Their experience of hope is shaped by how they navigate the “me” and the “I” within the self, under strong social pressure.

*It’s like this* (thinks), *it’s embarrassing, even to talk about it there with him. It’s uncomfortable, a stigmatized disease. Not bringing it up, not talking about syphilis also helps to cope.* (W1)

*It’s really hard, a lot of prejudice, guilt, shame, neglect, all together. And you’re there trying to survive. I talked a lot to myself, you know, things like, “Ignore it, stay strong, it won’t pass to the baby, endure it”. I think talking to myself helped me ignore what hurt.* (silence) *it’s almost just you and yourself. Me encouraging myself* (laughs). *Myself, like that. Because you can’t share it, it’s embarrassing* [...] (W4)

*I stay there, with my thoughts, thinking about everything I’m going through. I want to believe, I want to do everything so that it works out so that the disease doesn’t come to him. But it’s a lot of pressure, it feels like I don’t know anything, that they have to tell me how to do things because I got syphilis... I made a mistake* (silence). *I made a mistake and now I have to do everything as they say, as if I didn’t know how.* (W12)

After birth, in interactions with the child, women paid attention to signs they had been taught during prenatal care, such as “blindness”, “hearing problems”, and “mental problems”. When outcomes were favorable, they felt hopeful.

*She was born healthy and alert. I make sounds and see that she hears. She follows with her eyes, you know? She seems very alert... when breastfeeding, she looks at me, she seems fine, doesn’t seem to have anything* (reflective silence). *They put so much in our heads that I keep watching, but she seems very alert* (laughs). *In my mind, I almost went crazy thinking so many things.* (W8)

## DISCUSSION

The analyses allowed the identification that participants’ experience of hope is restricted to the particularized sphere, as there is a clear hoped-for object (the non-contamination of the unborn child) and investments toward its achievement, evidenced in the behavior of adhering to treatment and closely monitoring outcomes through diagnostic test results. The mothers maintained hope for non-transmission, which helped them cope with the adverse relational context, fears, concerns, and uncertainties. Hope is an existential component that shapes behaviors and relationships^(16)^ and, for these participants, was essential to cope with the stigma and fear associated with their condition, thereby attempting to regain control over their lives and their child’s health. Restricting relationships with healthcare professionals and disclosing the diagnosis to few members of their social network contributed to a less threatening context while awaiting outcomes.

Studies addressing the experience of women with GS have also identified, similarly to this study, adherence to syphilis treatment as a behavior among a significant portion of women^(19,20,21)^, as well as the strong presence of stigma and moral judgment in interactions with professionals^(19,20,21)^. Embarrassment in healthcare encounters involving sexually transmitted infections has been recurrently reported and must be overcome^(21)^. This study advances knowledge by incorporating the construct of hope to structure the analysis and deepen the understanding of these issues in the context of GS.

In light of the above, interventions aimed at hope in relation to the professional–patient relationship are relevant and necessary, especially empathetic presence^(1,22,23)^, relational grounding in respect and trust^(1,22)^, fostering an interactional context that encourages expression, welcoming concerns and feelings^(23,24)^, and active listening^(1)^. Furthermore, professionals need to be cautious in their language and terminology, as statements emphasizing potential harm may negatively impact women in terms of emotional suffering and hope.

Exposure to relationships marked by prejudice and stigma negatively impacted women’s perception of being supported and understood. This finding highlights the urgent need for positive experiences of support and welcoming care from professionals as essential for establishing and sustaining particularized hope. These include interventions aimed at hope and professional support to help women develop coping strategies for emotional suffering—expressed as guilt, fear, shame, and concerns^(21,22,23,24,25,26)^—as well as informational support using accessible language, with opportunities for clarification and dialogue. In addition to hope-focused interventions, the establishment of achievable goals^(1,24)^ and the use of praise for behaviors, efforts, and achievements^(1)^, such as adherence to treatment, are important.

Furthermore, updating informational content in alignment with the trajectory of the experience, its progression, and outcomes is important. However, participants reported receiving the same information and narrative throughout the entire gestational period. This aspect had a negative impact on their experience, including on hope. This finding contributes to the literature and represents an innovative aspect of this study. Hope is a process, and therefore professional support must also adapt throughout care^(16,25)^.

In the results, several dimensions of hope were evident, such as social support, interactions with healthcare professionals, spirituality, and belief in a better future, all essential for constructing meaning and a concrete and tangible object of hope.

Experiencing the diagnosis of GS and its treatment amid interactions in healthcare settings permeated by judgment and stigma generates a profound emotional impact^(21,24,26)^. Women reported feelings of shame and guilt^(24,26)^, which contrasts with the pursuit of hope. This dynamic reinforces how mental and emotional health are interconnected with perceptions of hope in this population. Hope is a predictor of emotional well-being. The lack of a welcoming and prejudice-free environment can be threatening, leading participants, whenever possible, to limit their exposure and their relationship with healthcare professionals. This is another innovative finding of this study.

Conversely, the findings allow us to state that building a safe and dialogical space, with education about syphilis and continuous support, can promote hope among women diagnosed with GS. Hope is strengthened when individuals feel they have access to properly guided information and engaged professional support. In the context of illness and healthcare, professionals play a distinctive role in providing informational and social support^(27)^, which was not evident among participants. Nevertheless, the reports demonstrated the driving force, maintenance, and direction that hope provided in coping with the situation. Thus, professionals can support hopeful thinking and help adjust expectations, goals, and objectives. In addition, they can help manage emotional burden and navigate challenges by using hope as a structuring element of care^(26)^. Developing competencies for managing hope in professional training is essential for advancing its effective incorporation into healthcare practice^(28)^.

Participants used hope to remain motivated to adhere to treatment for GS and to endure the waiting period, reflecting on performing well in their maternal role. The relationship between “willpower” and “waypower”^(5)^ was evident, as determination to follow treatment was connected to the goal of preventing transmission of the bacterium. Recognizing hope as an inner guiding force is to act within the context of hope^(1,2,3,16)^.

Various nursing bodies and organizations recognize hope as part of the profession’s scope. In the International Classification for Nursing Practice^(29)^, it is included, with interventions such as “Counseling about hope” and “Promoting hope”. It is also referenced in terms such as “hopelessness”, “despair”, and “support: providing confidence or hope to someone”^(29)^. Deliberately incorporating hope may enable prenatal, maternity, outpatient maternal-child, and Primary Health Care nurses to renew their practices in the context of GS and possibly transform the meaning of GS and congenital syphilis, improving relationships and healthcare outcomes. However, the relationship with professionals was shown to be a significant threat to hope in the prenatal context of women with GS, adding to other factors that already weaken and impact quality of care^(30)^.

In this sense, supporting the management of hope falls within the scope of nursing^(1,2,3)^, and one behavior that may guide professionals is to inquire about the focus of hope of the person receiving care, considering their particular experience and the factors that promote or threaten hope. Thus, in care dialogues, professionals should ask: what is hope for you? How can I help you with your hope?

Renewing healthcare in the context of GS involves attitudinal transformations and professional presence, considering the hope of women facing GS. This approach may transform interactional contexts and contribute to reducing the moral harassment reported, improving proximity and the quality of relationships, counteracting distancing, and impacting syphilis control. The management of hope as a professional practice was not identified in participants’ reports.

Although participants were adolescent and young women, black or mixed-race, these characteristics did not provide sufficient data for analyses related to hope. However, intersectionality is a reality^(13)^, and studies focusing on it in the experience of hope among these populations and others in situations of oppression are recommended. Furthermore, this aspect represents a limitation of the study, as do the exclusion criteria adopted and the fact that this was a secondary analysis of interviews focused on perceptions of healthcare.

## CONCLUSION

The findings allow us to state that participants’ hope is situated in the particularized sphere, with a clear hoped-for object: the non-contamination of the unborn child.

The experience of hope proved to be existential and processual in nature, occurring through investment in “promoters of hope” to counterbalance “threats to hope”. In this context, since interactions with healthcare professionals were significant threats, participants maintained only strictly necessary relationships in order to sustain hope. Support for emotional and informational needs was insufficient, and internalized reflection on the maternal role stood out. Protecting the child, their life, and development remained central and sustained the behavior of seeking and maintaining engagement with healthcare services.

## Data Availability

The entire dataset supporting the results of this study is available upon request to the corresponding author.
