# Supplementary material for: The experience of young women with gestational syphilis from the conceptual perspective of hope
Source: Rev Esc Enferm USP. 2026 Jul 24;60:e20250118. doi: 10.1590/1980-220X-REEUSP-2025-0118en (PMC13404300; doi:10.1590/1980-220X-REEUSP-2025-0118en)
Supplement: Translation

# Translation: Experiência de mulheres jovens com sífilis gestacional sob a ótica conceitual da esperança

**DOI:** 10.1590/1980-220X-REEUSP-2025-0118pt

**Authors:** Monika Wernet, Zaida Charepe, Constança Festas, Aline Oliveira Silveira, Patrícia Akari Nakao

**Affiliations:** 1 Universidade Federal de São Carlos, Departamento de Enfermagem, São Carlos, SP, Brasil.; 2 Universidade Católica Portuguesa, Faculdade de Ciências da Saúde e Enfermagem, Lisboa, Portugal.; 3 Universidade de Brasília, Departamento de Enfermagem, Brasília, DF, Brasil.; 4 Universidade Federal de São Carlos, Programa de Pós-graduação em Enfermagem, São Carlos, SP, Brasil.

**Keywords:** Sífilis, Atenção à Saúde, Esperança, Cuidado Pré-Natal, Relações Profissional-Paciente

## Abstract

**Objetivo:**

Compreender a esperança da mulher jovem diagnosticada com sífilis gestacional no contexto da atenção pré-natal.

**Método:**

Pesquisa qualitativa, embasada na análise temática, sob a ótica conceitual da esperança. Foi desenvolvida no estado de São Paulo com mulheres jovens diagnosticadas com sífilis gestacional no pré-natal por meio de entrevistas semiestruturadas e áudio gravadas realizadas entre julho de 2023 e abril de 2024.

**Resultados:**

Participaram do estudo 16 mulheres jovens, todas autodeclaradas pretas ou pardas, das quais quatro eram primíparas e a maioria tinha até nove anos de estudo. A vivência da esperança está retratada nas seguintes unidades temáticas: “Ameaças à esperança”, com os subtemas “Estigma e sofrimentos” e “Suporte informacional e social restritos”; e “Promotores da esperança”, com os subtemas “Proteção da criança” e “Preservação de si”.

**Conclusão:**

As mulheres enfrentaram, na atenção pré-natal, a dialética de contextos ameaçadores e promotores da esperança. Nesse sentido, destacaram-se, respectivamente, o suporte profissional lacunar às necessidades emocionais e informacionais e a clareza do objeto desejado (não contaminação da sua criança) associada à reflexão internalizada sobre o exercício de seu papel materno.

## INTRODUÇÃO

Há uma crescente produção de evidências sobre esperança na atenção à saúde e na enfermagem, todas assinalando os contributos do construto para o enfrentamento de situações adversas, ameaçadoras e/ou complexas. A esperança conduz à identificação de objetivo e ao sentido das experiências, sendo altamente pessoal^(1,2)^.

A incorporação da esperança na prática clínica favorece o cuidado holístico e integral^(1,2,3)^, centrado na pessoa, com reconhecimento da singularidade dos sofrimentos, dificuldades, necessidades e enfrentamentos^(2,3)^. Promove força transformadora, com expectativas positivas, motivação e disposição interior para o estabelecimento e alcance de objetivos^(2,3,4)^. O processo da esperança se relaciona à determinação (*willpower*) e aos comportamentos (*waypower*) para alcançar algo intencionado^(5)^, um objeto esperançado.

Diante da esperança, ocorrem comportamentos de apreciação e avaliação dinâmica e contínua da situação e dos recursos disponíveis em relação ao objeto desejado, com o estabelecimento de estratégias para alcançá-lo^(2,3,4,5,6,7)^. Isso promove resiliência e possibilita a superação de adversidades, com efeitos no conforto^(6)^, na qualidade de vida, no bem-estar^(7)^, na saúde^(6)^ e na vida. Altos níveis de esperança conduzem pessoas a assumirem, no presente, comportamentos direcionados a objetivos futuros^(6)^.

Ao pensar no encontro para processamento do cuidado em saúde e enfermagem, o comportamento do profissional é um determinante da esperança^(2,3,4)^, aspecto que implica assunção de compromissos com ela, considerando-a em suas intervenções^(3)^. As relações inter e intrapessoais estão entre os determinantes da esperança, delineando sua manifestação^(4,6)^.

As evidências relativas à esperança concentram-se no âmbito dos cuidados paliativos e adoecimentos crônico, abordando diferentes períodos de vida e as transições de saúde-doença^(1)^. Há lacunas de discussões dela diante do diagnóstico de sífilis, doença infectocontagiosa que se configura como problema e desafio à saúde pública mundial. A sífilis gestacional (SG), especificamente, está associada a um maior risco de aborto espontâneo, parto prematuro e ocorrência de sífilis congênita, todas ameaças à vida e à saúde, com desdobramentos para a morbimortalidade materno-infantil^(8,9)^.

De acordo com o Boletim Epidemiológico de 2024 do Ministério da Saúde do Brasil, em 2023, os casos de SG foram da ordem de 34 para cada 1.000 nascidos vivos, totalizando 86.111 notificações, um aumento de 11% em relação à taxa analisada do ano anterior^(9)^. Nesse cenário, adolescentes e jovens estiveram destacados como grupos populacionais de predomínio da ocorrência da doença^(9)^, sendo premente explorar discussões específicas a esses grupos.

O controle da SG tem sido um desafio. As evidências científicas e a abordagem clínica estão fortemente direcionadas pela epidemiologia do risco, com esforços máximos de diagnóstico precoce, tratamento oportuno da mulher e alcance de suas parcerias sexuais para diagnóstico e tratamento^(8,9)^, medidas importantes de intervenção. Apesar dos avanços, a doença revela reemergência no Brasil e mundo^(8,9,10)^, e reclama por inovações na atenção à saúde. Nessa direção, o acesso equitativo a serviços de saúde e de pré-natal de qualidade é apontado como um impacto na abordagem da situação^(11)^ e como garantia de direitos.

No Brasil, a história da doença sífilis e das campanhas de saúde dirigidas a ela abrange construções discursivas e simbólicas imersas em colocações dotadas de apontamentos morais, sobretudo quanto às práticas sexuais^(12)^. Ademais, identifica-se um forte acento no comportamento individual, estruturado em lógica biomédica, higienicista e sanitarista^(8,9,10)^.

Há premência de, nas respostas à reemergência da doença, transcender o enfoque acima assinalado, com consideração das questões sociais, culturais e subjetivas^(12,13)^, bem como de características geracionais. Rotulações sociais têm chances de se estender à percepção da pessoa de si e afetar enfrentamentos^(13)^, inclusive no âmbito da esperança.

O presente estudo indaga sobre a experiência da esperança de mulheres jovens diagnosticadas com SG no contexto da atenção pré-natal. Partiu-se do pressuposto de que esse recorte pode trazer indicativos de renovação das práticas de cuidado em saúde e de seus profissionais, contribuindo para um deslocamento de abordagem nos encontros voltados ao cuidado da doença, considerando as subjetividades e determinações sociais presentes no contexto. O objetivo foi de compreender a esperança da mulher jovem diagnosticada com SG no contexto da atenção pré-natal.

## MÉTODO

### Desenho do Estudo

Trata-se de estudo qualitativo, descritivo e exploratório, norteado pela ferramenta *COnsolidated criteria for REporting Qualitative research* ^(14)^, fundamentado pelo referencial metodológico da análise temática^(15)^ e pelo Modelo de Esperança de Dufault e Martocchio^(3)^. Foi desenvolvida análise secundária dos dados de pesquisa com mulheres jovens diagnosticadas com SG no pré-natal sobre a percepção do cuidado em saúde recebido.

A esperança abarca duas esferas, a generalizada e a particularizada, ambas com as mesmas seis dimensões: (1) afetiva, englobando as emoções e os sentimentos envolvidos com o objeto de esperança intencionado; (2) cognitiva, abarcando pensamentos e desejos vinculados ao objeto de esperança e envolvendo o processo de identificação dele e avaliação da realidade para sua consecução; (3) comportamental, remetendo à ação e à orientação para produzir o desejado; (4) afiliativa, vinculada às relações consigo, com os outros e com o meio social de inserção; (5) temporal, envolvendo o reconhecimento de que a esperança está dirigida ao futuro, mas é dependente de experiências do presente e do passado; e (6) contextual, traduzindo a consideração e a relevância do contexto social de inserção^(3)^. A esperança generalizada difere da particularizada por ser incerto o intencionado enquanto futuro. Na esperança particularizada, há um objeto de esperança estabelecido, para o qual são feitos investimentos. Essa última atua no enfrentamento construtivo^(3)^.

Ademais, a esperança é um processo existencial, delineando o sentir, o se comportar e o se relacionar consigo mesmo e com outros. Ela surge enquanto processo na e a partir da interação de quatro atributos: transcendência (alma da esperança); experiencial (dor da esperança); relacional (coração da esperança); e racional (mente da esperança)^(16)^.

### Local, População e Critérios de Seleção

A pesquisa foi desenvolvida em cidade brasileira situada no interior do estado de São Paulo. Os critérios de seleção para a participação no estudo foram ser mulher, ser emancipada na faixa etária de 15 aos 24 anos e ter diagnóstico de sífilis no período gestacional. Excluíram-se aquelas que não estavam em condições psíquicas de participar da entrevista narrativa.

O local de captação dos participantes foi a maternidade do centro-leste do estado de São Paulo, referência regional para gestação, parto e puerpério. Pertencente ao Departamento Regional de Saúde III, a maternidade registrou 1.991 partos em 2023 e 1.924 em 2024, dos quais cerca de 7% foram de bebês com SG. Desses, pouco mais da metade tinha entre 15 e 24 anos, de acordo com informações da gestão do serviço. Segundo o Instituto Brasileiro de Geografia e Estatística, em 2022, a população da cidade era de 254.857 habitantes, dos quais 6,73% eram mulheres entre 15 e 24 anos^(17)^.

### Coleta de Dados

A coleta de dados ocorreu entre julho de 2023 e abril de 2024 por meio de entrevistas áudio gravadas, realizadas nas casas das participantes. O convite para o estudo era feito pela enfermeira gerente da maternidade. Com o aceite, ela intermediava o contato com a equipe de pesquisa, obtendo o contato telefônico e solicitando autorização para compartilhá-lo. Em seguida, as pesquisadoras entravam em contato para agendar data e horário da entrevista. No dia da entrevista, elas retomavam os objetivos do estudo, confirmavam o aceite de participação e, então, procediam à assinatura do Termo de Consentimento Livre e Esclarecido (TCLE). Destaca-se que os excertos integrantes deste manuscrito foram identificados com a letra “M”, alusivos à palavra “mulher”, seguidos pelo número arábico representativo da ordem de sua entrada no estudo.

O primeiro momento da entrevista era direcionado à caracterização das mulheres em relação à idade, à situação conjugal, à cor, ao trimestre gestacional do diagnóstico da sífilis e aos dados gestacionais e de paridade. O segundo momento apresentou a colocação voltada à rememoração do “quando recebeu o diagnóstico da sífilis e como aconteceu e percebeu o cuidado”. Outras perguntas para aprofundar a percepção acerca do cuidado pré-natal recebido foram apresentadas de modo alinhado ao narrado pela participante.

As entrevistas tiveram duração de 18 a 35 minutos. Elas foram conduzidas pela última autora, pesquisadora em treinamento, sob supervisão e apoio da primeira autora, que tem experiência no desenvolvimento de estudos qualitativos com uso de entrevistas. Notas de campo foram feitas a respeito de detalhes contextuais para contribuir com a triangulação das fontes de obtenção dos dados e para as interpretações das entrevistas transcritas. Da mesma forma, as análises e os dados foram compartilhados nas reuniões do grupo de pesquisa *Hope2Care* para agregar perspectivas em relação aos dados e às análises. O referido grupo, liderado pela segunda autora deste manuscrito, abrange projetos de investigação, intervenção e formação em esperança nos processos de saúde-doença. Assim, os resultados parciais foram apresentados e discutidos em dois encontros do grupo, mantendo-se o anonimato das participantes.

O critério da saturação temática determinou o encerramento das entrevistas, isto é, o conjunto de dados obtidos e a sua análise alcançaram recorrência de códigos ou temas. Não surgiram novas informações ou relacionamentos entre eles surgiram na compreensão do objeto de estudo^(18)^.

### Análise dos Dados

Na direção de compreender a experiência da esperança da mulher, a reanálise das entrevistas do estudo primário foi guiada pelas colocações acerca das dimensões e esferas da esperança^(3)^ e de seu processo^(16)^.

O texto da transcrição das entrevistas foi analisado pela primeira autora, sob supervisão da segunda autora deste manuscrito. O processo analítico foi conduzido pela análise temática^(15)^, portanto estruturado em seis etapas. A primeira etapa consistiu em leituras repetidas para identificar significados e padrões associados ao objeto de estudo, a esperança no contexto do cuidado pré-natal de mulheres jovens com SG, com seleção de trechos representativos (segunda etapa, de seleção de elementos-chave). Esses trechos foram destacados considerando as dimensões da esperança^(3)^, seu processo e atributos^(16)^. Os esforços foram direcionados para a compreensão da esperança, de como ela se revelava e se conectava à existência da mulher, bem como das emoções, sentimentos e pensamentos dinamizados sob a percepção do contexto social que os continha. Além disso, a forma como esse todo se articulou com a consecução do objeto de esperança direcionou a seleção e a análise dos trechos. Esses trechos foram apreciados com o objetivo de compreender os significados das palavras e construções linguísticas. Ou seja, identificaram-se as palavras-chave, que foram posteriormente codificadas (terceira etapa da seleção dos códigos). Esses códigos foram integrados em temas (quarta etapa), e então ocorreram novos processos analíticos indutivos voltados para a identificação de padrões e relações entre eles (quinta etapa, da conceituação), encerrando o processo com o desenvolvimento do modelo conceitual, que corresponde à etapa final do método de análise temática. O modelo conceitual alcançado estruturou-se em dois temas principais, um relativo à dimensão promotora; e outro relativo à dimensão limitadora da experiência de esperança.

### Aspectos Éticos

A pesquisa seguiu os princípios deontológicos preconizado pelo Conselho Nacional de Saúde do Brasil para pesquisas envolvendo seres humanos. Foi aprovada por Comitê de Ética em Pesquisa com Seres Humanos, sob Parecer nº 6.200.614 e Certificado de Apresentação para Apreciação Ética nº 69634923.1.0000.5504. As participantes assinaram o TCLE.

## RESULTADOS

Vinte e três mulheres foram convidadas a integrar o estudo, das quais três não aceitaram participar por referirem que falar da situação gerava grande desconforto nelas. Das 20 mulheres que aceitaram, quatro não retornaram às duas tentativas de contato para agendamento da entrevista, sendo consideradas desistentes da participação. Assim, foram entrevistadas 16 mulheres com idade média de 18 anos. Duas eram emancipadas, e três tinham o histórico de reinfecção pelo *Treponema pallidum*. Todas as mulheres eram pretas ou pardas. Além disso, quatro eram primíparas; seis eram secundíparas; 11 tinham até nove anos de estudo; e uma era mãe solo.

A vivência da esperança da mulher jovem diagnosticada com SG no contexto do cuidado em saúde ocorre a partir do manejo dialético de aspectos ameaçadores e promotores da esperança, conforme Quadros 1 e 2.

**Quadro 1:** Códigos e subtemas do tema “Ameaças à esperança” – São Carlos, SP, Brasil, 2025.

Códigos iniciais	Códigos intermediários	Subtema
Percepções de estigma	Estigma e preconceito	Estigma e sofrimentos
Vivências de violências		
Não exposição de si		
Desconfortos	Sobrecarga emocional	
Culpa		
Medos e preocupações		
Dúvidas e incipiência de conhecimentos	Desconhecimento	Suporte informacional e social restritos
Uso da internet		
Partilha da condição e apoio social	Rede de apoio social limitada	
Solidão		
Tema: Ameaças à esperança		

**Quadro 2:** Códigos e subtemas do tema “Promotores da esperança” – São Carlos, SP, Brasil, 2025.

Códigos iniciais	Códigos intermediários	Subtema
Credo na proteção divina	Credos	Proteção da criança
Credo em bons desfechos		
Exames	Adesão às recomendações profissionais	
Tratamento		
Ida às consultas		
Interação com a criança	Sinalizações a si	Preservação de si
Pensamento sobre si no exercício do papel materno		
Rompimento de relações	Respeitando seus limites	
Reflexões sobre humilhações		
Tema: Promotores da esperança		

No contexto das relações e comunicações com os profissionais de saúde durante o pré-natal, elas vivenciaram as principais ameaças à sua esperança. Porém, elas precisaram se expor a essas ameaças para alcançar o objeto de sua esperança: a não contaminação da criança que estavam gestando. Eram os profissionais que lhes davam a garantia do tratamento para a SG e o acesso aos exames diagnósticos. Ao reafirmarem seu envolvimento com a terapêutica voltada à sífilis, sentiram que estavam protegendo sua criança, o que foi central para a projeção e o fortalecimento da esperança. Os temas “Ameaças à esperança” e “Promotores da esperança”, com seus respectivos subtemas, detalham a experiência de esperança da mulher jovem diagnosticada com SG no contexto da atenção à saúde e estão sintetizados na Figura 1.

**Figura 1:** Síntese gráfica do modelo conceitual “Experiência de esperança da mulher jovem com sífilis gestacional na atenção à saúde”. São Carlos, SP, Brasil, 2025.

*Tema: Ameaças à esperança*

*Subtema: Estigma e sofrimentos*

A esperança da mulher, mãe da criança exposta ao *Treponema pallidum* na gestação, edifica-se em contexto relacional imerso em comunicações que sugerem preconceito e determinam sentimentos como vergonha, mal-estar e desconforto.

Na percepção da mulher, o profissional, ao interagir com o símbolo “SG”, aciona o significado de dano à criança e passa a enfatizar, no decorrer da interação, que a bactéria é transmissível por via uteroplacentária e pode ter consequências drásticas para a criança, sendo seu dever realizar o tratamento prescrito. Nesse contexto relacional, as comunicações dos profissionais (verbais, não verbais, paralinguagem) veiculam símbolos de emissão de juízo de valor. A recorrência e a intensidade dessas comunicações reverberam em violência moral, conduzindo a mulher a buscar não se expor nas interações com os profissionais.

*É muito julgamento. Eles* (profissionais da maternidade) *cochicham entre eles e você percebe tudo.* [...] *eu lembro de quando fiz o teste e deu* (positivo para sífilis). *A enfermeira arregalou o olho, já foi falando se eu sabia o que era, que era doença de sexo, que eu tinha que me cuidar, que era nova, que ia prejudicar ele* (criança gestada). *Falou muito. Fiquei envergonhada, queria correr dali; eu só olhava para baixo.* (M16)

As interações no contexto do cuidado em saúde geram desconforto, de modo que ponderaram interromper as idas aos serviços para não precisarem se expor a elas.

*No pré-natal, já tive que passar por um monte de preconceito da sífilis como doença de quem sai com todo mundo.* [...] *eu peguei do pai dela. Dói estas coisas,* [...] *dá vergonha, muita vergonha.* [...] *tem hora que é tanto que penso em não vir; venho querendo não vir.* (M5)

*Olham para você e já vem preconceito da “sífilis”* (pensa). [...] *é bem ruim o clima da conversa e dá mal-estar.* (silêncio) *Daí você entra e sai sempre o mais quieta. Responde o necessário e corre dali. Eu sempre fico agitada para vir.* [...] *eu não gosto de vir não, pois já sei como vai ser*. (M10)

Destarte, sentiram culpa por pensar que sua criança esteve ou está em risco de danos pela SG e sofreram com isso.

*A primeira gestação é um momento único ali. E aí uma doença dessa! Nossa Senhora, Nossa Senhora, pegou bem. Mas eu fiz o tratamento, todos os exames. Fiz os exames bonitinho, a gente não pode deixar, né? Não tem como deixar, é a nossa saúde, não só a nossa saúde como a bebê.* [...] *chorei e como chorei... sozinha. Choro até hoje de pensar que pode ficar cego e retardado por culpa minha.* (M13)

O processo de analisar as evidências à luz do que vivencia imputou sobrecarga emocional e esgotamento moral, outro elemento que ameaçou a esperança.

### Subtema – Suporte Informacional e Social Restritos

Estar submetida a relações nos serviços de saúde permeadas de preconceito reduz as chances de a mulher ampliar e qualificar as informações que detém acerca da sífilis. Elas não se sentiram encorajadas a apresentar dúvidas, com medo de serem expostas a falas que as julguem. Todas as participantes deste estudo revelaram ter lacunas de conhecimento sobre a sífilis e afirmaram recorrer à internet para saná-las. Elas evitam apresentá-las nas interações com os profissionais por temerem o desconforto. Dessa forma, o conhecimento para compreender sua situação e gerar esperança é construído com base em informações restritas sobre a sífilis.

*Eu não tinha conhecimento* (da doença)*. Até hoje, eu não sei assim certinho. Quando falaram* (da doença), *eu fiquei assustada. Eu pesquisei, fui pesquisar o que era, daí li que, se faz o tratamento, fica tudo bem. Daí eu fiquei tranquila, pois eu fiz o tratamento, só que, até hoje, eu não entendo, assim, se o tratamento tira a doença do corpo, se a doença nunca sai do corpo. Parece que fica, né? Nunca vai embora, né? Na criança também?* (M10)

Além disso, conscientes do significado socialmente difundido da sífilis, elas contavam para poucas pessoas de sua rede social sobre sua situação. Quase tomaram isso como um segredo entre elas, geralmente compartilhado apenas com o companheiro e/ou alguém próximo da família, principalmente a mãe. Inclusive, uma participante buscou colocar o evento em “esquecimento”:

*Eu fiquei com medo de ter passado para ela* (criança), *mas eu fiz seis doses do remédio. Fez o exame de sangue e deu reagente, daí repetiu de novo e já não deu, deu que só tinha um pouquinho, então decidimos* (ela e o companheiro) *esquecer o assunto. Não vamos mais ir atrás de nada e não contamos para ninguém* [...] *a gente decidiu não contar para ninguém e esquecer, apagar* [...] (M1)

Diante do conjunto exposto acima, a vivência é quase que solitária, tendo a si, em suas reflexões internalizadas, como a principal parceria no manejo de dúvidas, medos e preocupações. Ressalta-se que para uma participante, não saber para além do que os profissionais lhe informaram foi estratégia para favorecer o enfrentamento.

*Fiquei com medo, com receio... mexe com o psicológico da gente. Fiquei com raiva, fiquei com medo de poder afetar alguma coisa nele. Não procurei mais nada, para não me abalar mais.* (M4)

### Tema: Promotores da Esperança

A vivência da esperança sustenta-se na proteção de si e da criança. Para isso, buscam preservar a si e garantir o melhor desempenho do papel de mãe.

### Subtema: Proteção da Criança

As interações junto aos profissionais de saúde, desde o diagnóstico da SG, promoveram o entendimento de que a sífilis é avaliada por meio de exames e de o tratamento ser o ato que garante a “cura” ou a não contaminação da criança gestada pela bactéria. Assim, todas as participantes fizeram o tratamento e realizaram os exames conforme as indicações dos profissionais, que foram experienciados como símbolos de cuidado materno. Nesse contexto, diante de incertezas, clamavam pela proteção divina.

[...] *ficou mais forte o não passar para ela... isto que eu valorizei, pensei nela, em não passar para ela.* [...] *daí já fui pensando nela e vendo tudo o que eu tinha que fazer, e disseram ser o tratamento e fazer os exames para ver se deu certo. E fiz. Então, o que ajudou foi pensar nela e pensar que ia dar certo, pois eu estava fazendo tudo que estavam falando.* (M1)

*Fiz tudo certinho, como mandaram. Espero que dê certo dele não sofrer e de não ter nada.* (M7)

*Faltou uns dias para dar aquele tempo do fim do remédio e o nascimento dela. Estou meio nervosa se vai dar certo. Desanimei um pouco, fiz tanto esforço, aguentei aquelas injeções todas, doídas demais. Não pode acontecer de ter a doença. Se Deus quiser, não vai. Deus vai proteger, eu acredito Nele* [...] (M11)

Entre as mulheres que já haviam vivenciado a sífilis em gestação anterior, a interação com essa criança contribuía com o enfrentamento. O fato de deterem uma experiência prévia contribuiu com as tomadas de decisão para a situação atual. Ademais, o credo na proteção divina potencializava sua esperança.

*Acreditar em Deus, rezei muito. Toda noite eu rezava pedindo pela saúde dele. Eu sou muito crente. Eu vou toda semana na igreja e sempre pedia pela saúde dele. E me deixa ver que me ajudava* (pensa)... *acho que olhar para meu outro filho e ver que deu certo com ele.* (M2)

### Subtema: Preservação de Si

A sobrecarga emocional do vivido nas relações com os profissionais instaurou um processo intenso de reflexões internalizadas, as quais reafirmavam à mulher seus atos para minimizar a transmissão da bactéria da sífilis à criança, do quanto teve esforços em garantir o que estava ao seu alcance. Essas conversas regularam tomadas de decisão relativas à exposição social, sobretudo pelo estigma associado à doença. Buscavam, assim, garantir certa proteção a si para tolerar as humilhações, as culpas e as preocupações, a fim de sustentar sua esperança. Sua vivência de esperança é moldada a partir de como dinamizou a fase do “mim” e do “eu” em seu *self*, sob forte coação do socialmente (im)posto.

*É assim* (pensa), *dá vergonha, até de falar ali com ele. É chato, doença malvista. Não tocar no assunto, não ficar falando da sífilis também ajuda a enfrentar.* (M1)

*É difícil mesmo, muito preconceito, culpa, vergonha, descaso, tudo junto. E você ali tentando sobreviver. Eu conversava muito comigo, sabe, coisa assim: “Ignora, força, não vai passar para o bebê, aguenta”. Acho que conversar comigo me ajudava a ignorar o que doía.* (silêncio) *É quase você e você. Eu dando força para mim* (ri). *Eu mesma assim. Porque não dá para dividir, dá vergonha,* [...] (M4)

*Fico lá, com minhas caraminholas, pensando tudo que estou passando. Quero acreditar, quero fazer tudo para que dê certo, para que não venha a doença para ele. Só que é muita pressão, parece que não sei, que eles têm que dizer como tenho que fazer pois peguei sífilis... errei* (silêncio). *Errou e agora tem que fazer como mandam, como se eu não soubesse fazer.* (M12)

Após ao nascimento, nas interações com a criança, atentaram-se aos símbolos que lhe foram ensinados no pré-natal, a exemplo de “cegueira”, “problema de audição” e “problema mental”. Quando os desfechos foram bons, sentiram-se esperançosos.

*Nasceu bem e esperta. Faço sonzinho e vejo que ouve. Busca com o olhinho, sabe? Parece bem esperta... no mamar, olha para mim, é esperta, não parece ter nada* (silêncio reflexivo). *Falam tanto na cabeça da gente que estou de olho, parece que é bem esperta* (ri). *Na minha cabeça, eu já pirei de tanto pensar coisas.* (M8)

## DISCUSSÃO

As análises permitiram identificar que a experiência de esperança das participantes se restringe à esfera particularizada, já que há um objeto de esperança claro (a não contaminação da criança gestada) e investimentos para que ele se concretize, evidenciados no comportamento de se tratar e acompanhar de forma expectante o desfecho a partir dos resultados dos exames diagnósticos. As mães mantiveram a esperança na não transmissão, o que as ajudou a enfrentar o contexto relacional adverso, os medos, as preocupações e as incertezas. A esperança é componente existencial, delineadora de comportamentos e relações^(16)^ e, para essas participantes, foi essencial para enfrentar o estigma e o medo associados à sua condição, tentando, assim, recuperar o controle das suas vidas e da saúde de sua criança. Restringir relações com os profissionais de saúde e revelar a condição diagnóstica para poucos da sua rede social favoreceram um contexto menos ameaçador na espera do desfecho.

Estudos voltados à experiência de mulheres diante da SG assinalaram, assim como este estudo, a adesão ao tratamento da sífilis como comportamento de parcela significativa das mulheres^(19,20,21)^ e a forte presença do estigma e do julgamento moral nas relações com os profissionais^(19,20,21)^. O constrangimento no encontro de produção do cuidado às infecções sexualmente transmissíveis está recorrentemente denunciado e precisa ser transposto^(21)^. Este estudo avança o conhecimento ao trazer o construto esperança para estruturar as análises e adensar compreensão dessas questões no âmbito da SG.

Diante do apontamento acima, intervenções voltadas à esperança relativas ao relacionamento com o profissional são pertinentes e necessárias, com destaque à presença empática^(1,22,23)^, à ancoragem relacional no respeito e na confiança^(1,22)^, à edificação do contexto interacional promotor da expressão e ao acolhimento de preocupações, sentimentos^(23,24)^ e escuta ativa^(1)^. Ademais, os profissionais precisam ser cautelosos na linguagem e nos termos, cabendo assinalar o impacto negativo que colocações relativas aos danos trazem à mulher em termos de sofrimentos e impacto emocional e na esperança.

A submissão a relações marcadas pelo preconceito e estigma impactou negativamente a percepção das mulheres de se sentirem apoiadas e compreendidas. Essa análise remete-nos à premência de experiências positivas de suporte e acolhimento junto a profissionais como significativas para o estabelecimento e sustentação da esperança particularizada. São intervenções direcionadas à esperança e ao suporte profissional para o encontro de estratégias de enfrentamento do sofrimento emocional, expresso em culpa, medo, vergonha e preocupações^(21,22,23,24,25,26)^, assim como suporte informacional em linguagem acessível, com oportunidade de esclarecimentos e diálogo. Além das intervenções para apoiar a esperança, existe o estabelecimento de metas alcançáveis^(1,24)^ e o uso de elogios para comportamentos, esforços e conquistas^(1)^, como a adesão ao tratamento.

Destarte, a atualização da pauta informativa, alinhada à trajetória da vivência, ao seu percurso e aos desfechos, é importante, mas as participantes denunciaram a manutenção das mesmas informações e o enredo informativo ao longo de todo o período gestacional. Esse aspecto exerceu um impacto negativo sobre a experiência delas, inclusive no que diz respeito à esperança. Esse destaque é uma contribuição deste estudo e inova as evidências existentes. A esperança é processual e, com isso, ao suporte profissional também está posto o ajustamento ao longo do cuidado^(16,25)^.

Nos resultados, as várias dimensões da esperança estiveram evidenciadas, como o apoio social, as interações com os profissionais de saúde, a espiritualidade e a crença em um futuro melhor, todas essenciais para a construção de um sentido e de um objeto de esperança concreto e tangível.

Vivenciar o diagnóstico da SG e seu tratamento em meio a interações nos equipamentos de saúde, imersas em julgamento e estigmatização, gera um impacto emocional profundo^(21,24,26)^. As mulheres relataram sentimentos de vergonha e culpa^(24,26)^, o que contrasta com a procura da esperança. Essa dinâmica reforça também, nesta população, como a saúde mental e emocional está interligada às percepções de esperança. A esperança é um preditor de bem-estar emocional. A falta de um ambiente acolhedor e livre de preconceito pode ser ameaçadora, fazendo com que as participantes, sempre que possível, limitem sua exposição e sua relação com o profissional de saúde. Essa é outra evidência inovadora apontada por este estudo.

Em contraponto, as colocações permitem-nos afirmar que a construção de um espaço seguro e de diálogo, com educação sobre a sífilis e suporte contínuo, pode promover a esperança entre as mulheres diagnosticadas com SG. A esperança é reforçada quando as pessoas sentem que têm acesso a informações corretamente orientadas e um suporte profissional interessado. No contexto do adoecimento e da atenção à saúde, os profissionais de saúde detêm um papel diferenciado em relação ao suporte informacional e social^(27)^, aspecto não evidenciado entre as participantes. Apesar disso, os relatos permitiram visualizar a propulsão, a sustentação e o direcionamento providos pela esperança ao enfrentamento da situação. Desse modo, o profissional oferece suporte ao pensamento esperançoso e ao ajuste de expectativas, metas e objetivos. Além disso, ele pode ajudar a gerir a carga emocional e transitar pelos desafios ao ter a esperança como um estruturante da intervenção^(26)^. Desenvolver competências para o manejo da esperança na formação profissional é essencial para avanços na efetiva incorporação dela na prática dos cuidados de saúde^(28)^.

As participantes utilizaram a esperança para se manterem motivadas a cumprir a terapia para a SG e para vivenciar a espera, refletindo sobre uma boa performance no papel materno. A relação entre “*willpower*” e “*waypower*”^(5)^ foi evidenciada, sendo que a determinação em seguir o tratamento se conecta com a necessidade de alcançar o objetivo de que a transmissão da bactéria não ocorra. Reconhecer a esperança como uma força interior e orientadora é atuar no contexto da esperança^(1,2,3,16)^.

Várias instâncias e entidades representativas da enfermagem referendam a esperança como escopo da profissão. Na Classificação Internacional da Prática de Enfermagem^(29)^ ela está contemplada, e entre as intervenções direcionadas a ela constam: “Aconselhar sobre esperança” e “Promover esperança”. No entanto, ela também é lembrada em termos como “desespero”, “desolação” e “apoiar: dar confiança ou esperança a alguém”^(29)^. Assumir deliberadamente a esperança pode favorecer o enfermeiro pré-natalista, de maternidade, de ambulatórios maternoinfantis e da Atenção Primária à Saúde a renovar suas práticas no contexto da SG e, quiçá, a modificar o sentido e o significado da SG e da sífilis congênita, qualificando relações e alcances da atenção à saúde. Porém, a relação com o profissional revelou ser um importante fator de ameaça à esperança no contexto do pré-natal de mulheres com SG, somando-se a outros que já o fragilizam e impactam a sua qualidade^(30)^.

Nesse sentido, o suporte à gestão da esperança está no escopo do enfermeiro^(1,2,3)^, e um comportamento que pode conduzir o profissional a desempenhar esse papel é indagar sobre o foco da esperança do demandante de cuidados, considerando a vivência particular apresentada e os fatores promotores e ameaçadores da esperança tangível. Assim, nos diálogos de cuidado, cabe ao profissional indagar: o que é esperança para você? Como posso ajudá-lo(a) na sua esperança?

A renovação da atenção à saúde no contexto da SG envolve transformações atitudinais e de presença dos profissionais, considerando a esperança das mulheres que enfrentam a SG. Esse posicionamento pode vir a modificar os contextos interacionais e favorecer a redução do assédio moral denunciado, com efeitos sobre a proximidade e a qualidade da relação, contrapondo distanciamentos e tendo impactos no controle da sífilis. Não se evidenciou nos relatos o manejo da esperança como prática profissional.

Apesar de as participantes serem mulheres adolescentes e jovens, pretas e pardas, essas determinações não tiveram dados que permitissem análises relativas à esperança. Porém, a interseccionalidade é uma realidade^(13)^, e estudos que a coloquem como foco primário na experiência de esperança dessas pessoas e de outras que vivem em situação de opressão são sugeridos. Ademais, este aspecto é um limite deste estudo, assim como o critério de exclusão adotado e o fato de ser uma análise secundária de entrevistas direcionadas à percepção sobre o cuidado em saúde.

## CONCLUSÃO

Os achados permitem afirmar que a esperança das participantes está na esfera particularizada, com objeto esperançado evidente: a não contaminação da criança gestada.

A vivência da esperança revelou-se de caráter existencial e processual, ocorrendo sob o investimento nos “promotores da esperança” para contrapor as “ameaças à esperança”. Nesse contexto, uma vez que as interações com os profissionais de saúde são importante ameaças, as participantes manterem relações estritamente necessárias para poderem seguir esperançando. O suporte às necessidades emocionais e informacionais foi insuficiente, e a reflexão internalizada sobre o papel materno destacou-se. Proteger a criança, sua vida e seu desenvolvimento esteve no horizonte e sustentou o comportamento de buscar e manter-se em acompanhamento na atenção à saúde.

## Data Availability

Todo o conjunto de dados que dá suporte aos resultados deste estudo está disponível mediante solicitação ao autor correspondente.

# Translation: La experiencia de mujeres jóvenes con sífilis gestacional desde la perspectiva conceptual de la esperanza

**DOI:** 10.1590/1980-220X-REEUSP-2025-0118es

**Authors:** Monika Wernet, Zaida Charepe, Constança Festas, Aline Oliveira Silveira, Patrícia Akari Nakao

**Affiliations:** 1 Universidade Federal de São Carlos, Departamento de Enfermagem, São Carlos, SP, Brasil.; 2 Universidade Católica Portuguesa, Faculdade de Ciências da Saúde e Enfermagem, Lisboa, Portugal.; 3 Universidade de Brasília, Departamento de Enfermagem, Brasília, DF, Brasil.; 4 Universidade Federal de São Carlos, Programa de Pós-graduação em Enfermagem, São Carlos, SP, Brasil.

**Keywords:** Sífilis, Atención a la Salud, Esperanza, Atención Prenatal, Relaciones Profesional-Paciente

## Abstract

**Objetivo:**

Comprender la esperanza de las jóvenes diagnosticadas con sífilis gestacional en el contexto de la atención prenatal.

**Método:**

Investigación cualitativa, basada en análisis temático, desde la perspectiva conceptual de la esperanza. Se desarrolló en el estado de São Paulo con jóvenes diagnosticadas con sífilis gestacional durante la atención prenatal mediante entrevistas semiestructuradas y grabaciones de audio realizadas entre julio de 2023 y abril de 2024.

**Resultados:**

Participaron dieciséis mujeres jóvenes, todas identificadas como negras o mestizas, cuatro de ellas madres primerizas, y la mayoría contaba con hasta nueve años de escolaridad. La experiencia de la esperanza se representa en las siguientes unidades temáticas: “Amenazas a la esperanza”, con los subtemas “Estigma y sufrimiento” y “Falta de información y apoyo social”; y “Factores que promueven la esperanza”, con los subtemas “Protección infantil” y “Autoconservación”.

**Conclusión:**

En la atención prenatal, las mujeres se enfrentaron a una dialéctica de contextos amenazantes y esperanzadores. En este sentido, destacaron, respectivamente, el apoyo profesional insuficiente para sus necesidades emocionales e informativas, y la claridad del objetivo deseado (la no contaminación de su hijo) asociada a la reflexión internalizada sobre el ejercicio de su rol materno.

## INTRODUCCIÓN

Existe una creciente producción de evidencia sobre la esperanza en la atención de la salud y en la enfermería, toda la cual señala las contribuciones de este constructo para el manejo de situaciones adversas, amenazantes y/o complejas. La esperanza conduce a la identificación de objetivos y al sentido de las experiencias, siendo altamente personal^(1,2)^.

La incorporación de la esperanza en la práctica clínica favorece la atención holística e integral^(1,2,3)^, centrada en la persona, con reconocimiento de la singularidad de los sufrimientos, las dificultades, las necesidades y los enfrentamientos^(2,3)^. Promueve una fuerza transformadora, con expectativas positivas, motivación y disposición interior para el establecimiento y el logro de objetivos^(2,3,4)^. El proceso de la esperanza se relaciona con la determinación (*willpower*) y los comportamientos (*waypower*) para alcanzar algo intencionado^(5)^, un objeto esperado.

Ante la esperanza, se producen comportamientos de apreciación y evaluación dinámica y continua de la situación y de los recursos disponibles en relación con el objeto deseado, con el establecimiento de estrategias para alcanzarlo^(2,3,4,5,6,7)^. Esto promueve la resiliencia y permite superar las adversidades, con efectos en el bienestar^(6)^, la calidad de vida, la salud^(6)^ y la vida. Los altos niveles de esperanza llevan a las personas a adoptar, en el presente, comportamientos orientados a objetivos futuros^(6)^.

Al pensar en el encuentro para el procesamiento de la atención en salud y enfermería, el comportamiento del profesional es un determinante de la esperanza^(2,3,4)^, aspecto que implica asumir compromisos con ella, considerándola en sus intervenciones^(3)^. Las relaciones inter e intrapersonales se encuentran entre los determinantes de la esperanza, delineando su manifestación^(4,6)^.

Las evidencias relativas a la esperanza se concentran en el ámbito de los cuidados paliativos y las enfermedades crónicas, abordando diferentes etapas de la vida y las transiciones entre salud y enfermedad^(1)^. Existen lagunas en los debates sobre ella ante el diagnóstico de sífilis, una enfermedad infecciosa que se configura como un problema y un desafío para la salud pública mundial. La sífilis gestacional (SG), específicamente, se asocia con un mayor riesgo de aborto espontáneo, parto prematuro y aparición de sífilis congénita, todas ellas amenazas para la vida y la salud, con consecuencias para la morbilidad y mortalidad materno-infantil^(8,9)^.

Según el Boletín Epidemiológico de 2024 del Ministerio de Salud de Brasil, en 2023 los casos de SG fueron del orden de 34 por cada 1.000 nacidos vivos, con un total de 86.111 notificaciones, lo que representa un aumento del 11 % con respecto a la tasa analizada del año anterior^(9)^. En este contexto, las adolescentes y las jóvenes se destacaron como los grupos poblacionales con mayor prevalencia de la enfermedad^(9)^, por lo que es urgente explorar debates específicos para estos grupos.

El control de la SG ha sido un desafío. La evidencia científica y el enfoque clínico están fuertemente orientados por la epidemiología del riesgo, con esfuerzos máximos para el diagnóstico precoz, el tratamiento oportuno de la mujer y la identificación de sus parejas sexuales para su diagnóstico y tratamiento^(8,9)^, medidas de intervención importantes. A pesar de los avances, la enfermedad muestra un resurgimiento en Brasil y en el mundo^(8,9,10)^, y exige innovaciones en la atención de salud. En este sentido, el acceso equitativo a servicios de salud y de atención prenatal de calidad se señala como un factor de impacto en el enfoque de la situación^(11)^ y como garantía de derechos.

En Brasil, la historia de la sífilis y de las campañas de salud dirigidas a ella abarca construcciones discursivas y simbólicas inmersas en planteamientos dotados de connotaciones morales, sobre todo en lo que respecta a las prácticas sexuales^(12)^. Además, se identifica un fuerte énfasis en el comportamiento individual, estructurado en una lógica biomédica, higienista y sanitarista^(8,9,10)^.

Existe la necesidad de que, en las respuestas al resurgimiento de la enfermedad, se trascienda el enfoque anteriormente señalado, tomando en cuenta las cuestiones sociales, culturales y subjetivas^(12,13)^, así como las características generacionales. Las estigmatizaciones sociales pueden extenderse a la percepción que la persona tiene de sí misma y afectar a sus estrategias de afrontamiento^(13)^, incluso en el ámbito de la esperanza.

El presente estudio indaga sobre la experiencia de la esperanza de las mujeres jóvenes diagnosticadas con SG en el contexto de la atención prenatal. Se partió de la premisa de que este enfoque puede aportar indicios de renovación de las prácticas de atención en salud y de sus profesionales, contribuyendo a un cambio de enfoque en los encuentros orientados al cuidado de la enfermedad, considerando las subjetividades y determinaciones sociales presentes en el contexto. El objetivo fue comprender la esperanza de las mujeres jóvenes diagnosticadas con SG en el contexto de la atención prenatal.

## MÉTODO

### Diseño del Estudio

Se trata de un estudio cualitativo, descriptivo y exploratorio, guiado por la herramienta *COnsolidated criteria for REporting Qualitative research* ^(14)^, basado en el marco metodológico del análisis temático^(15)^ y en el Modelo de Esperanza de Dufault y Martocchio^(3)^. Se llevó a cabo un análisis secundario de los datos de investigación con mujeres jóvenes diagnosticadas con SG en la etapa prenatal sobre la percepción de la atención de salud recibida.

La esperanza abarca dos esferas, la generalizada y la particularizada, ambas con las mismas seis dimensiones: (1) afectiva, que engloba las emociones y los sentimientos relacionados con el objeto de esperanza intencionado; (2) cognitiva, que abarca pensamientos y deseos vinculados al objeto de esperanza e implica el proceso de identificación del mismo y la evaluación de la realidad para su consecución; (3) conductual, que remite a la acción y a la orientación para producir lo deseado; (4) afiliativa, vinculada a las relaciones consigo mismo, con los demás y con el entorno social de inserción; (5) temporal, que implica el reconocimiento de que la esperanza se dirige hacia el futuro, pero depende de experiencias del presente y del pasado; y (6) contextual, que traduce la consideración y la relevancia del contexto social de inserción^(3)^. La esperanza generalizada difiere de la particularizada porque lo deseado es incierto en cuanto al futuro. En la esperanza particularizada, hay un objeto de esperanza establecido, en el que se realizan inversiones. Esta última actúa en el enfrentamiento constructivo^(3)^.

Además, la esperanza es un proceso existencial que perfila el sentir, el comportarse y el relacionarse consigo mismo y con los demás. Surge como proceso en y a partir de la interacción de cuatro atributos: trascendencia (alma de la esperanza); experiencial (dolor de la esperanza); relacional (corazón de la esperanza); y racional (mente de la esperanza)^(16)^.

### Lugar, Población y Criterios de Selección

La investigación se llevó a cabo en una ciudad brasileña situada en el interior del estado de São Paulo. Los criterios de selección para participar en el estudio fueron ser mujer, ser mayor de edad, tener entre 15 y 24 años y haber recibido un diagnóstico de sífilis durante el embarazo. Se excluyó a aquellas que no se encontraban en condiciones psíquicas para participar en la entrevista narrativa.

El lugar de reclutamiento de las participantes fue la maternidad del centro-este del estado de São Paulo, centro de referencia regional para el embarazo, el parto y el puerperio. Perteneciente al Departamento Regional de Salud III, la maternidad registró 1.991 partos en 2023 y 1.924 en 2024, de los cuales alrededor del 7 % fueron de bebés con SG. De estos, poco más de la mitad tenía entre 15 y 24 años, según información de la administración del servicio. Según el Instituto Brasileño de Geografía y Estadística, en 2022 la población de la ciudad era de 254 857 habitantes, de los cuales el 6,73 % eran mujeres de entre 15 y 24 años^(17)^.

### Recolección de Datos

La recolección de datos se llevó a cabo entre julio de 2023 y abril de 2024 mediante entrevistas grabadas en audio, realizadas en los hogares de las participantes. La invitación al estudio la realizaba la enfermera gerente de la maternidad. Una vez aceptada, ella facilitaba el contacto con el equipo de investigación, obtenía el número de teléfono y solicitaba autorización para compartirlo. A continuación, las investigadoras se ponían en contacto para programar la fecha y la hora de la entrevista. El día de la entrevista, volvían a explicar los objetivos del estudio, confirmaban la aceptación de la participación y, a continuación, procedían a la firma del Término de Consentimiento Libre e Informado (TCLI). Cabe destacar que los extractos que componen este manuscrito se identificaron con la letra «M», en alusión a la palabra «mujer», seguida del número arábigo que representa el orden de su inclusión en el estudio.

La primera parte de la entrevista se centró en la caracterización de las mujeres en cuanto a edad, estado civil, color, trimestre gestacional en el momento del diagnóstico de sífilis y datos gestacionales y de paridad. La segunda parte se centró en la rememoración de «cuándo recibió el diagnóstico de sífilis, cómo ocurrió y cómo percibió la atención». Se plantearon otras preguntas para profundizar en la percepción sobre la atención prenatal recibida, en consonancia con lo narrado por la participante.

Las entrevistas tuvieron una duración de entre 18 y 35 minutos. Fueron realizadas por la última autora, investigadora en formación, bajo la supervisión y el apoyo de la primera autora, quien cuenta con experiencia en el desarrollo de estudios cualitativos mediante entrevistas. Se tomaron notas de campo sobre detalles contextuales para contribuir a la triangulación de las fuentes de obtención de datos y a las interpretaciones de las entrevistas transcritas. Del mismo modo, los análisis y los datos se compartieron en las reuniones del grupo de investigación *Hope2Care* para agregar perspectivas en relación con los datos y los análisis. Dicho grupo, liderado por la segunda autora de este manuscrito, abarca proyectos de investigación, intervención y formación en esperanza en los procesos de saludenfermedad. Así, los resultados parciales se presentaron y discutieron en dos reuniones del grupo, manteniendo el anonimato de las participantes.

El criterio de saturación temática determinó el cierre de las entrevistas, es decir, el conjunto de datos obtenidos y su análisis alcanzaron la recurrencia de códigos o temas. No surgieron nuevas informaciones ni relaciones entre ellos en la comprensión del objeto de estudio^(18)^.

### Análisis de los Datos

Con el fin de comprender la experiencia de la esperanza de la mujer, el reanálisis de las entrevistas del estudio primario se guió por las consideraciones sobre las dimensiones y esferas de la esperanza^(3)^ y su proceso^(16)^.

El texto de la transcripción de las entrevistas fue analizado por la primera autora, bajo la supervisión de la segunda autora de este manuscrito. El proceso analítico se llevó a cabo mediante el análisis temático^(15)^, por lo que se estructuró en seis etapas. La primera etapa consistió en lecturas repetidas para identificar significados y patrones asociados al objeto de estudio, la esperanza en el contexto de la atención prenatal de mujeres jóvenes con SG, con la selección de fragmentos representativos (segunda etapa, de selección de elementos clave). Estos fragmentos se destacaron considerando las dimensiones de la esperanza^(3)^, su proceso y sus atributos^(16)^. Los esfuerzos se dirigieron a la comprensión de la esperanza, de cómo se revelaba y se conectaba con la existencia de la mujer, así como de las emociones, los sentimientos y los pensamientos dinamizados bajo la percepción del contexto social que los contenía. Además, la forma en que todo este conjunto se articulaba con la consecución del objeto de esperanza orientó la selección y el análisis de los fragmentos. Estos fragmentos se evaluaron con el objetivo de comprender los significados de las palabras y las construcciones lingüísticas. Es decir, se identificaron las palabras clave, que posteriormente se codificaron (tercera etapa de selección de códigos). Estos códigos se integraron en temas (cuarta etapa), y luego se llevaron a cabo nuevos procesos analíticos inductivos orientados a la identificación de patrones y relaciones entre ellos (quinta etapa, de la conceptualización), concluyendo el proceso con el desarrollo del modelo conceptual, que corresponde a la etapa final del método de análisis temático. El modelo conceptual obtenido se estructuró en dos temas principales, uno relativo a la dimensión promotora y otro relativo a la dimensión limitadora de la experiencia de la esperanza.

### Aspectos Éticos

La investigación siguió los principios deontológicos recomendados por el Consejo Nacional de Salud de Brasil para investigaciones que involucran a seres humanos. Fue aprobada por el Comité de Ética en Investigación con Seres Humanos, bajo el Dictamen n.º 6.200.614 y el Certificado de Presentación para Apreciación Ética n.º 69634923.1.0000.5504. Las participantes firmaron el TCLI.

## RESULTADOS

Se invitó a participar en el estudio a veintitrés mujeres, de las cuales tres no aceptaron participar porque indicaron que hablar de la situación les generaba un gran malestar. De las 20 mujeres que aceptaron, cuatro no respondieron a los dos intentos de contacto para programar la entrevista, por lo que se las consideró desistentes de la participación. Así, se entrevistó a 16 mujeres con una edad media de 18 años. Dos eran independientes, y tres tenían antecedentes de reinfección por *Treponema pallidum*. Todas las mujeres eran de raza negra o mestiza. Además, cuatro eran primíparas; seis eran secundíparas; 11 tenían hasta nueve años de estudios; y una era madre soltera.

La experiencia de la esperanza en las mujeres jóvenes diagnosticadas con SG en el contexto de la atención sanitaria se basa en la gestión dialéctica de los aspectos que amenazan y los que fomentan la esperanza, tal y como se muestra en los Cuadros 1 y 2.

**Cuadro 1:** Códigos y subtemas del tema «Amenazas a la esperanza» – São Carlos, SP, Brasil, 2025

Códigos iniciales	Códigos intermedios	Subtema
Percepciones de estigma	Estigma y prejuicio	Estigma y sufrimiento
Experiencias de violencia		
No revelación de la propia identidad		
Malestar	Sobrecarga emocional	
Culpa		
Miedos y preocupaciones		
Dudas y conocimientos incipientes	Desconocimiento	Apoyo informativo y social limitado
Uso de Internet		
Compartir la situación y apoyo social	Red de apoyo social limitada	
Soledad		
Tema: Amenazas a la esperanza		

**Cuadro 2:** Códigos y subtemas del tema «Promotores de la esperanza» – São Carlos, SP, Brasil, 2025

Códigos iniciales	Códigos intermedios	Subtema
Creo en la protección divina	Creencias	Protección de la infancia
Creo en los buenos resultados		
Exámenes	Cumplimiento de las recomendaciones profesionales	
Tratamiento		
Asistencia a las consultas		
Interacción con el niño	Señales para ti	Autocuidado
Reflexión sobre uno mismo en el ejercicio del papel maternal		
Ruptura de relaciones	Respetando tus límites	
Reflexiones sobre las humillaciones		
Tema: Promotores de la esperanza		

En el contexto de las relaciones y la comunicación con los profesionales de la salud durante el periodo prenatal, ellas vivieron las principales amenazas a su esperanza. Sin embargo, tuvieron que exponerse a esas amenazas para alcanzar el objetivo de su esperanza: que el niño que llevaban en su vientre no se contagiara.

Eran los profesionales quienes les daban la garantía del tratamiento para la SG y el acceso a los exámenes diagnósticos. Al reafirmar su compromiso con la terapia dirigida a la sífilis, sintieron que estaban protegiendo a su hijo, lo cual fue fundamental para la proyección y el fortalecimiento de la esperanza. Los temas «Amenazas a la esperanza» y «Promotores de la esperanza», con sus respectivos subtemas, detallan la experiencia de esperanza de la mujer joven diagnosticada con SG en el contexto de la atención de salud y se sintetizan en la Figura 1.

**Figura 1:** Síntesis gráfica del modelo conceptual «Experiencia de esperanza de la mujer joven con sífilis gestacional en la atención de salud». São Carlos, SP, Brasil, 2025.

*Tema: Amenazas a la esperanza*

*Subtema: Estigma y sufrimiento*

La esperanza de la mujer, madre del niño expuesto al *Treponema pallidum* durante el embarazo, se construye en un contexto relacional inmerso en comunicaciones que sugieren prejuicios y determinan sentimientos como la vergüenza, el malestar y la incomodidad.

En la percepción de la mujer, el profesional, al interactuar con el símbolo «SG», activa el significado de daño al niño y pasa a enfatizar, en el transcurso de la interacción, que la bacteria es transmisible por vía uteroplacentaria y puede tener consecuencias drásticas para el niño, siendo su deber realizar el tratamiento prescrito. En este contexto relacional, las comunicaciones de los profesionales (verbales, no verbales, paralenguaje) transmiten símbolos que emiten juicios de valor. La recurrencia y la intensidad de estas comunicaciones se traducen en violencia moral, lo que lleva a la mujer a tratar de no exponerse en las interacciones con los profesionales.

*Es mucho juicio. Ellos* (los profesionales de la maternidad) *cuchichean entre ellos y tú te das cuenta de todo.* [...] *Recuerdo cuando me hice la prueba y dio* (positivo para sífilis). *La enfermera abrió mucho los ojos, empezó a decirme si sabía lo que era, que era una enfermedad de transmisión sexual, que tenía que cuidarme, que era joven, que le haría daño a él* (al bebé que llevaba en el vientre). *Habló mucho. Me sentí avergonzada, quería salir corriendo de allí; solo miraba hacia abajo.* (M16)

Las interacciones en el contexto de la atención en salud generan incomodidad, por lo que consideraron dejar de acudir a los servicios para no tener que exponerse a ellas.

*En el control prenatal, ya tuve que pasar por un montón de prejuicios sobre la sífilis como una enfermedad de quienes salen con todo el mundo.* [...] *Me la contagié del papá de ella. Estas cosas duelen,* [...] *da vergüenza, mucha vergüenza.* [...] *Hay momentos en que es tanto que pienso en no venir; vengo sin ganas de venir.* (M5)

*Te miran y ya viene el prejuicio de la “sífilis”* (piensa). [...] *el ambiente de la conversación es muy malo y te hace sentir incómoda.* (silencio) *Entonces entras y sales siempre lo más callada posible. Respondes lo necesario y sales corriendo de ahí. Siempre me pongo nerviosa por venir.* [...] *no me gusta venir, porque ya sé cómo va a ser*. (M10)

Así, sintieron culpa por pensar que su bebé estuvo o está en riesgo de sufrir daños por la SG y sufrieron por ello.

*El primer embarazo es un momento único. ¡Y ahí una enfermedad así! Nuestra Señora, Nuestra Señora, me afectó mucho. Pero me hice el tratamiento, todos los exámenes. Me hice las pruebas como se debe, no podemos dejarlo pasar, ¿no? No hay forma de dejarlo pasar, es nuestra salud, no solo la nuestra sino la del bebé.* [...] *lloré y cómo lloré... sola.*

*Lloro hasta hoy al pensar que puede quedarse ciego y con retraso por mi culpa.* (M13)

El proceso de analizar la evidencia a la luz de lo que vive le causó una sobrecarga emocional y agotamiento moral, otro elemento que amenazó la esperanza.

### Subtema – Apoyo Informativo y Social Restringido

Estar sometida a relaciones en los servicios de salud impregnadas de prejuicios reduce las posibilidades de que la mujer amplíe y mejore la información que tiene sobre la sífilis. No se sintieron animadas a plantear dudas, por miedo a ser expuestas a comentarios que las juzgaran. Todas las participantes de este estudio revelaron tener lagunas de conocimiento sobre la sífilis y afirmaron recurrir a Internet para subsanarlas. Evitan plantearlas en las interacciones con los profesionales por temor a la incomodidad. De esta manera, el conocimiento para comprender su situación y generar esperanza se construye a partir de información limitada sobre la sífilis.

*No tenía conocimiento* (de la enfermedad)*. Hasta hoy, no lo sé con certeza. Cuando hablaron* (de la enfermedad), *me asusté. Investigué, fui a buscar qué era, y leí que, si se hace el tratamiento, todo queda bien. Entonces me quedé tranquila, porque hice el tratamiento, solo que, hasta hoy, no entiendo, así, si el tratamiento elimina la enfermedad del cuerpo, si la enfermedad nunca sale del cuerpo. Parece que se queda, ¿no? Nunca se va, ¿no? ¿En el niño también?* (M10)

Además, conscientes del significado socialmente difundido de la sífilis, contaban a pocas personas de su círculo social sobre su situación. Casi siempre lo consideraban un secreto entre ellas, que por lo general solo compartían con su pareja y/o alguien cercano de la familia, principalmente la madre. De hecho, una participante intentó dejar el asunto en el «olvido»:

*Me dio miedo haberle contagiado a ella* (la niña), *pero me tomé seis dosis del medicamento. Se hizo el análisis de sangre y dio positivo, luego lo repitió y ya no dio, dio que solo tenía un poquito, así que decidimos* (ella y su pareja) *olvidar el asunto. Ya no vamos a investigar nada y no se lo contamos a nadie* [...] *decidimos no contárselo a nadie y olvidarlo, borrarlo* [...] (M1)

Ante lo expuesto anteriormente, la experiencia es casi solitaria, teniéndose a sí misma, en sus reflexiones internalizadas, como principal aliada en el manejo de dudas, miedos y preocupaciones. Cabe destacar que, para una participante, no saber más allá de lo que le informaron los profesionales fue una estrategia para facilitar el afrontamiento.

*Me quedé con miedo, con temor... nos afecta psicológicamente. Me enojé, tuve miedo de poder afectarlo de alguna manera. No busqué nada más, para no perturbarme más.* (M4)

### Tema: Promotores de la Esperanza

La experiencia de la esperanza se sustenta en la protección de sí misma y del niño. Para ello, buscan preservarse a sí mismas y garantizar el mejor desempeño del papel de madre.

### Subtema: Protección del Niño

Las interacciones con los profesionales de la salud, desde el diagnóstico de la SG, promovieron la comprensión de que la sífilis se evalúa mediante exámenes y que el tratamiento es el acto que garantiza la «curación» o la no contaminación del feto por la bacteria. Así, todas las participantes se sometieron al tratamiento y se realizaron los exámenes según las indicaciones de los profesionales, quienes fueron experimentados como símbolos de cuidado materno. En este contexto, ante las incertidumbres, clamaban por la protección divina.

[...] *se hizo más fuerte el no contagiarla... esto es lo que valoré, pensé en ella, en no contagiarla.* [...] *entonces ya empecé a pensar en ella y a ver todo lo que tenía que hacer, y me dijeron que era el tratamiento y hacerme los exámenes para ver si había funcionado. Y lo hice. Entonces, lo que me ayudó fue pensar en ella y pensar que iba a salir bien, porque estaba haciendo todo lo que me decían.* (M1)

*Hice todo bien, como me dijeron. Espero que salga bien, que él no sufra y que no tenga nada.* (M7)

*Faltan unos días para que termine el tratamiento y nazca ella. Estoy un poco nerviosa por si sale bien. Me desanimé un poco, hice tanto esfuerzo, aguanté todas esas inyecciones, que dolían mucho. No puede ser que tenga la enfermedad. Si Dios quiere, no la tendrá. Dios la protegerá, yo creo en Él* [...] (M11)

Entre las mujeres que ya habían padecido sífilis en un embarazo anterior, la interacción con ese niño contribuía a afrontar la situación. El hecho de tener una experiencia previa contribuyó a la toma de decisiones para la situación actual. Además, la fe en la protección divina potenciaba su esperanza.

*Creer en Dios, recé mucho. Todas las noches rezaba pidiendo por su salud. Soy muy creyente. Voy todas las semanas a la iglesia y siempre pedía por su salud. Y me da la sensación de que me ayudaba* (piensa)... *creo que mirar a mi otro hijo y ver que con él salió bien.* (M2)

### Subtema: Autoprotección

La sobrecarga emocional de lo vivido en las relaciones con los profesionales instauró un intenso proceso de reflexiones internalizadas, las cuales reafirmaban a la mujer en sus actos para minimizar la transmisión de la bacteria de la sífilis al niño, de cuánto se había esforzado por garantizar lo que estaba a su alcance. Estas conversaciones regularon la toma de decisiones relativas a la exposición social, sobre todo por el estigma asociado a la enfermedad. Buscaban, así, garantizar cierta protección para sí mismas a fin de tolerar las humillaciones, las culpas y las preocupaciones, con el objetivo de mantener viva su esperanza. Su experiencia de esperanza se moldea a partir de cómo dinamizó la fase del «yo» y del «mí» en su *self*, bajo una fuerte coacción de lo socialmente (im)puesto.

*Es así* (piensa), *da vergüenza, hasta hablar allí con él. Es molesto, una enfermedad mal vista. No tocar el tema, no estar hablando de la sífilis también ayuda a enfrentarlo.* (M1)

*Es realmente difícil, mucho prejuicio, culpa, vergüenza, indiferencia, todo junto. Y tú allí tratando de sobrevivir. Me hablaba mucho a mí misma, ya sabes, cosas así: «Ignóralo, ámate, no se le va a contagiar al bebé, aguanta». Creo que hablar conmigo misma me ayudaba a ignorar lo que dolía.* (silencio) *Es casi tú y tú. Yo dándome fuerzas a mí misma* (ríe). *Yo misma así. Porque no se puede compartir, da vergüenza,* [...] (M4)

*Me quedo ahí, con mis preocupaciones, pensando en todo lo que estoy pasando. Quiero creer, quiero hacer todo lo posible para que salga bien, para que no le pase la enfermedad a él. Pero es mucha presión, parece que no sé, que ellos tienen que decirme cómo tengo que hacerlo porque me contagié de sífilis... me equivoqué* (silencio). *Te equivocaste y ahora tienes que hacer lo que te dicen, como si no supieras hacerlo.* (M12)

Tras el nacimiento, en las interacciones con la niña, prestaron atención a los símbolos que les habían enseñado en el prenatal, como «ceguera», «problema de audición» y «problema mental». Cuando los resultados fueron buenos, se sintieron esperanzados.

*Nació bien y lista. Hago un ruidito y veo que escucha. Busca con la miradita, ¿sabes? Parece muy lista... al mamar, me mira, es lista, no parece tener nada* (silencio reflexivo). *Nos hablan tanto en la cabeza de que estoy atenta, parece que es muy lista* (ríe). *En mi cabeza, ya me volví loca de tanto pensar cosas.* (M8)

## DISCUSIÓN

Los análisis permitieron identificar que la experiencia de esperanza de las participantes se restringe a la esfera particularizada, ya que hay un objeto de esperanza claro (la no contaminación del feto) e inversiones para que se concrete, evidenciadas en el comportamiento de cuidarse y acompañar con expectación el desenlace a partir de los resultados de los exámenes diagnósticos. Las madres mantuvieron la esperanza en la no transmisión, lo que las ayudó a enfrentar el contexto relacional adverso, los miedos, las preocupaciones y las incertidumbres. La esperanza es un componente existencial, que define comportamientos y relaciones^(16)^ y, para estas participantes, fue esencial para enfrentar el estigma y el miedo asociados a su condición, intentando así recuperar el control de sus vidas y de la salud de su hijo. Limitar las relaciones con los profesionales de la salud y revelar el diagnóstico solo a unos pocos de su red social favoreció un contexto menos amenazante a la espera del desenlace.

Los estudios centrados en la experiencia de las mujeres ante la SG señalaron, al igual que este estudio, la adherencia al tratamiento de la sífilis como comportamiento de una parte significativa de las mujeres^(19,20,21)^ y la fuerte presencia del estigma y el juicio moral en las relaciones con los profesionales^(19,20,21)^. La incomodidad en el encuentro de la prestación de cuidados para las infecciones de transmisión sexual es denunciada recurrentemente y debe superarse^(21)^. Este estudio amplía el conocimiento al incorporar el constructo de la esperanza para estructurar los análisis y profundizar la comprensión de estas cuestiones en el ámbito de la SG.

Ante lo señalado anteriormente, las intervenciones orientadas a la esperanza en relación con la relación con el profesional son pertinentes y necesarias, destacando la presencia empática^(1,22,23)^, el anclaje relacional en el respeto y la confianza^(1,22)^, la construcción de un contexto interactivo que promueva la expresión y la acogida de preocupaciones, sentimientos^(23,24)^ y la escucha activa^(1)^. Además, los profesionales deben ser cautelosos en el lenguaje y los términos, cabe señalar el impacto negativo que las afirmaciones relativas a los daños tienen en la mujer en términos de sufrimiento, impacto emocional y esperanza.

La sumisión a relaciones marcadas por el prejuicio y el estigma afectó negativamente la percepción de las mujeres de sentirse apoyadas y comprendidas. Este análisis nos remite a la urgencia de experiencias positivas de apoyo y acogida junto a los profesionales como significativas para el establecimiento y el mantenimiento de la esperanza particularizada. Se trata de intervenciones dirigidas a la esperanza y al apoyo profesional para encontrar estrategias de afrontamiento del sufrimiento emocional, expresado en culpa, miedo, vergüenza y preocupaciones^(21,22,23,24,25,26)^, así como apoyo informativo en un lenguaje accesible, con oportunidad de aclaraciones y diálogo. Además de las intervenciones para apoyar la esperanza, existe el establecimiento de metas alcanzables^(1,24)^ y el uso de elogios por comportamientos, esfuerzos y logros^(1)^, como la adherencia al tratamiento.

De este modo, la actualización de la información, alineada con la trayectoria de la experiencia, su recorrido y los resultados, es importante, pero las participantes denunciaron el mantenimiento de la misma información y el mismo guion informativo a lo largo de todo el período gestacional. Este aspecto tuvo un impacto negativo en su experiencia, incluso en lo que respecta a la esperanza. Este punto es una contribución de este estudio e innova las evidencias existentes. La esperanza es procesual y, por lo tanto, el apoyo profesional también debe adaptarse a lo largo de la atención^(16,25)^.

En los resultados, se evidenciaron las diversas dimensiones de la esperanza, como el apoyo social, las interacciones con los profesionales de la salud, la espiritualidad y la creencia en un futuro mejor, todas esenciales para la construcción de un sentido y de un objeto de esperanza concreto y tangible.

Vivir el diagnóstico de la SG y su tratamiento en medio de interacciones en los centros de salud, inmersos en el juicio y la estigmatización, genera un profundo impacto emocional^(21,24,26)^. Las mujeres relataron sentimientos de vergüenza y culpa^(24,26)^, lo que contrasta con la búsqueda de esperanza. Esta dinámica refuerza también, en esta población, cómo la salud mental y emocional está interrelacionada con las percepciones de esperanza. La esperanza es un predictor del bienestar emocional. La falta de un entorno acogedor y libre de prejuicios puede resultar amenazante, lo que hace que las participantes, siempre que sea posible, limiten su exposición y su relación con el profesional de la salud. Esta es otra evidencia innovadora señalada por este estudio.

Por el contrario, los comentarios nos permiten afirmar que la construcción de un espacio seguro y de diálogo, con educación sobre la sífilis y apoyo continuo, puede promover la esperanza entre las mujeres diagnosticadas con SG. La esperanza se refuerza cuando las personas sienten que tienen acceso a información correctamente orientada y a un apoyo profesional interesado. En el contexto de la enfermedad y la atención de la salud, los profesionales de la salud desempeñan un papel diferenciado en relación con el apoyo informativo y social^(27)^, aspecto no evidenciado entre las participantes. A pesar de ello, los relatos permitieron visualizar el impulso, el sustento y la orientación que proporciona la esperanza para afrontar la situación. De este modo, el profesional ofrece apoyo al pensamiento esperanzador y al ajuste de expectativas, metas y objetivos. Además, puede ayudar a gestionar la carga emocional y a superar los desafíos al utilizar la esperanza como un elemento estructurante de la intervención^(26)^. Desarrollar competencias para el manejo de la esperanza en la formación profesional es esencial para avanzar en su incorporación efectiva a la práctica de la atención de salud^(28)^.

Las participantes utilizaron la esperanza para mantenerse motivadas a cumplir con la terapia para la SG y para vivir la espera, reflexionando sobre un buen desempeño en el papel materno. Se evidenció la relación entre «*willpower*» y «*waypower*»^(5)^, ya que la determinación de seguir el tratamiento se conecta con la necesidad de alcanzar el objetivo de que no ocurra la transmisión de la bacteria. Reconocer la esperanza como una fuerza interior y orientadora es actuar en el contexto de la esperanza^(1,2,3,16)^.

Varias instancias y entidades representativas de la enfermería respaldan la esperanza como ámbito de la profesión. En la Clasificación Internacional de la Práctica de Enfermería^(29)^ está contemplada, y entre las intervenciones dirigidas a ella figuran: «Asesorar sobre la esperanza» y «Promover la esperanza». Sin embargo, también se menciona en términos como «desesperación», «desolación» y «apoyar: dar confianza o esperanza a alguien»^(29)^. Asumir deliberadamente la esperanza puede favorecer que el personal de enfermería prenatal, de maternidad, de consultorios materno-infantiles y de Atención Primaria de Salud renueve sus prácticas en el contexto de la SG y, tal vez, modifique el sentido y el significado de la SG y de la sífilis congénita, mejorando las relaciones y el alcance de la atención de salud. Sin embargo, la relación con el profesional se reveló como un importante factor de amenaza para la esperanza en el contexto prenatal de las mujeres con SG, sumándose a otros que ya la fragilizan e impactan su calidad^(30)^.

En este sentido, el apoyo a la gestión de la esperanza está dentro del ámbito del enfermero^(1,2,3)^, y un comportamiento que puede llevar al profesional a desempeñar este papel es indagar sobre el foco de la esperanza de la persona que solicita cuidados, considerando la experiencia particular presentada y los factores que promueven y amenazan la esperanza tangible. Así, en los diálogos de atención, corresponde al profesional preguntar: ¿qué es la esperanza para usted? ¿Cómo puedo ayudarle en su esperanza?

La renovación de la atención de salud en el contexto de la SG implica transformaciones en las actitudes y la presencia de los profesionales, teniendo en cuenta la esperanza de las mujeres que enfrentan la SG. Esta postura puede llegar a modificar los contextos interaccionales y favorecer la reducción del acoso moral denunciado, con efectos sobre la cercanía y la calidad de la relación, contrarrestando el distanciamiento y teniendo impactos en el control de la sífilis. En los relatos no se evidenció el manejo de la esperanza como práctica profesional.

A pesar de que las participantes eran adolescentes y mujeres jóvenes, de raza negra y mestiza, estas características no contaban con datos que permitieran realizar análisis relacionados con la esperanza. Sin embargo, la interseccionalidad es una realidad^(13)^, y se sugieren estudios que la sitúen como foco principal en la experiencia de esperanza de estas personas y de otras que viven en situaciones de opresión. Además, este aspecto constituye una limitación de este estudio, al igual que el criterio de exclusión adoptado y el hecho de que se trate de un análisis secundario de entrevistas orientadas a la percepción sobre la atención en salud.

## CONCLUSIÓN

Los hallazgos permiten afirmar que la esperanza de las participantes se encuentra en la esfera particularizada, con un objeto esperado evidente: la no contaminación del feto.

La vivencia de la esperanza se reveló de carácter existencial y procesual, ocurriendo bajo la inversión en los «promotores de la esperanza» para contrarrestar las «amenazas a la esperanza». En este contexto, dado que las interacciones con los profesionales de la salud son amenazas importantes, las participantes mantienen relaciones estrictamente necesarias para poder seguir esperando. El apoyo a las necesidades emocionales e informativas fue insuficiente, y se destacó la reflexión internalizada sobre el papel materno. Proteger al niño, su vida y su desarrollo estuvo en el horizonte y sustentó el comportamiento de buscar y mantenerse en seguimiento en la atención de salud.

## Data Availability

El conjunto de datos que respaldan las conclusiones de este estudio está disponible previa solicitud al autor correspondiente.
